# Heterogeneous fibroblasts contribute to fibrotic scar formation after spinal cord injury in mice and monkeys

**DOI:** 10.1038/s41467-024-50564-x

**Published:** 2024-07-27

**Authors:** Xiaoyu Xue, Xianming Wu, Yongheng Fan, Shuyu Han, Haipeng Zhang, Yuting Sun, Yanyun Yin, Man Yin, Bing Chen, Zheng Sun, Shuaijing Zhao, Qi Zhang, Weiyuan Liu, Jiaojiao Zhang, Jiayin Li, Ya Shi, Zhifeng Xiao, Jianwu Dai, Yannan Zhao

**Affiliations:** 1 State Key Laboratory of Molecular Developmental Biology, Institute of Genetics and Developmental Biology, Chinese Academy of Sciences, Beijing, 100101 China; 2 University of Chinese Academy of Sciences, Beijing, 100101 China; 3 Tianjin Key Laboratory of Biomedical Materials, Institute of Biomedical Engineering, Chinese Academy of Medical Sciences & Peking Union Medical College, Tianjin, 300192 China

**Keywords:** Diseases of the nervous system, Cellular neuroscience, Spinal cord diseases

## Abstract

Spinal cord injury (SCI) leads to fibrotic scar formation at the lesion site, yet the heterogeneity of fibrotic scar remains elusive. Here we show the heterogeneity in distribution, origin, and function of fibroblasts within fibrotic scars after SCI in mice and female monkeys. Utilizing lineage tracing and single-cell RNA sequencing (scRNA-seq), we found that perivascular fibroblasts (PFs), and meningeal fibroblasts (MFs), rather than pericytes/vascular smooth cells (vSMCs), primarily contribute to fibrotic scar in both transection and crush SCI. Crabp2 + /Emb+ fibroblasts (CE-F) derived from meninges primarily localize in the central region of fibrotic scars, demonstrating enhanced cholesterol synthesis and secretion of type I collagen and fibronectin. In contrast, perivascular/pial Lama1 + /Lama2+ fibroblasts (LA-F) are predominantly found at the periphery of the lesion, expressing laminin and type IV collagen and functionally involved in angiogenesis and lipid transport. These findings may provide a comprehensive understanding for remodeling heterogeneous fibrotic scars after SCI.

## Abstract (web summary)

The heterogeneous characters of fibrotic scars after spinal cord injury remains unclear. Here, the authors show the heterogeneous distribution, source and function of meningeal fibroblasts and perivascular fibroblasts in fibrotic scars.

## Introduction

Fibrosis, defined as the massive deposition of extracellular matrix (ECM) by myofibroblasts, usually occurs in response to damage and inflammation in peripheral organs and the central nervous system (CNS)^1,2^. After spinal cord injury (SCI), two types of scars are generated at the lesion site, astrocyte scars consisting of proliferating GFAP+ astrocytes and fibrotic scars filled with ECM-producing myofibroblasts^3–5^. Fibrotic scars are considered barriers to axon regeneration, and thereby contribute to permanent loss of sensory and motor function^6^. Strategies have been developed to ablate or decrease scarring to improve functional recovery. Interestingly, complete ablation of the fibrotic scar often results in a large cavity that is detrimental to damage repair, but partially reducing scarring enhances axon regeneration and functional recovery^7–13^. This suggests that the compositions and functions of fibrotic scar tissues are heterogeneous after SCI.

Previously, it was generally believed that fibrotic scar formation is restricted to meningeal fibroblasts (MFs) after penetrating SCI in which the meninges are torn^14^. Subsequently, it was found that non-penetrating CNS injuries in which meninges remain intact can also lead to the formation of fibrotic scars^6^. Perivascular fibroblasts (PFs) have been shown to be the primary source of fibrotic scarring in non-penetrating injuries, whereas MFs are also involved in the formation of fibrotic scars after penetrating SCI^2,15^. However, other studies have argued that fibrotic scars originate primarily from type A pericytes in both penetrating and non-penetrating SCI^7,16^. Although lineage tracing strategies have been employed to investigate the origin of fibrotic scar tissue after SCI, the origin of myofibroblasts is still controversial due to the absence of recognized marker genes that distinguish these cells. The difference between meningeal and perivascular fibroblasts in both penetrating and non-penetrating SCI remains under debate.

Here, we aimed to reveal the composition and function of cells in fibrotic scar tissue by answering the following questions. Are the compositions and sources of fibrotic scars in penetrating and non-penetrating SCI different? Are the contributions of pericytes/ vascular smooth cells (vSMCs) and fibroblasts to fibrosis different? Are there functionally heterogeneous subpopulations of fibroblasts in fibrotic scars? Considering that pericytes/vSMCs and fibroblasts are two distinct types of cells^17^ that both express PDFGRβ^18–20^, we took used of PDFGRβ-CreER in combination with the R26-TdTomato (TdT) reporter to examine the heterogeneity of pericytes/vSMCs and fibroblasts in fibrotic scar tissue after SCI. Immunostaining and single-cell RNA sequencing (scRNA-seq) results showed that PDGFRβ+ cells were distributed in the perivascular spaces and meninges of the intact spinal cord. ScRNA-seq of spinal cords from mice and monkeys revealed that PDGFRβ+ cells were pericytes/vSMCs and fibroblasts (MFs and PFs) with specific gene signatures. Combined with lineage tracing of fibroblasts (by Col1a2-CreER::R26-TdT mice), pericytes/vSMCs (by Myh11-CreER::R26-TdT and NG2-CreER::R26-TdT mice) and meningeal fibroblasts (by Crabp2-CreER:: R26-TdT mice), we found that the fibrotic scars formed after transection SCI and crush SCI were derived from PFs and MFs rather than pericytes/vSMCs. Crabp2+/Emb+ fibroblasts (CE-F) derived from meninges and perivascular/pial Lama1+/Lama2+ fibroblasts (LA-F) exhibited different spatial distributions and functional heterogeneity in the lesion sites of both mice and monkeys. The heterogeneous characteristics of fibrotic scars revealed in our study might help to develop strategies for remodeling fibrotic scars after SCI.

## Results

### PDGFRβ+ cells are recruited to SCI lesions, with transection SCI showing higher recruitment compared to crush SCI

**Fig. 1:**
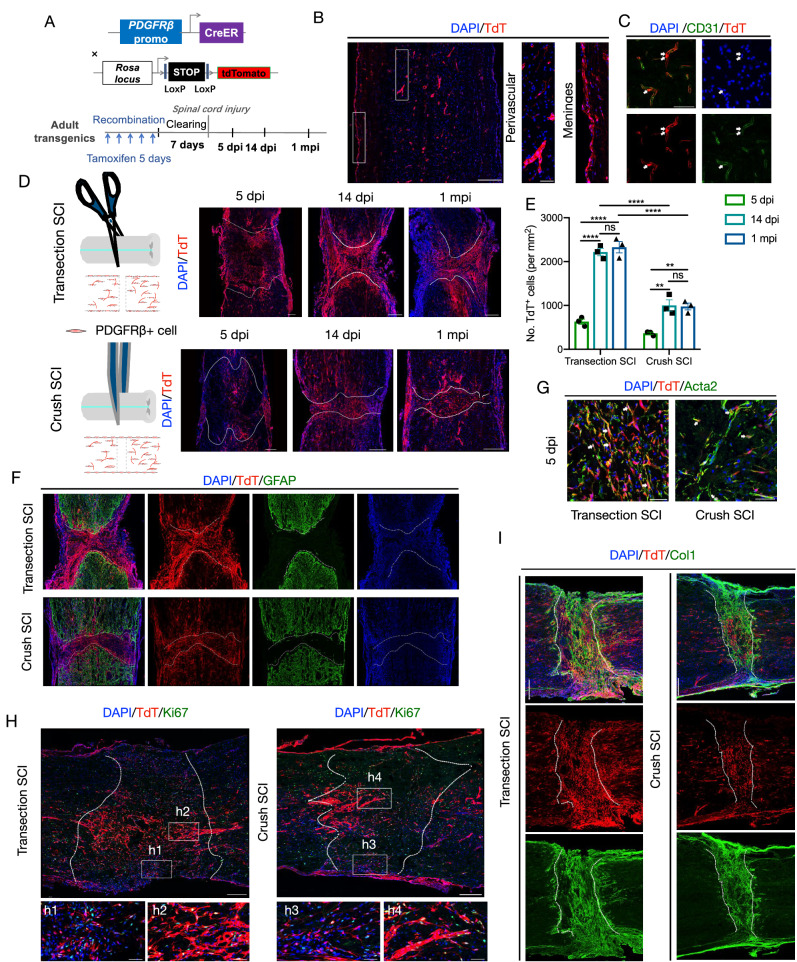
More PDGFRβ+ cells are recruited to the lesion core following transection spinal cord injury compared to crush spinal cord injury. **A** Schematic showing the protocol tracing PDGFRβ progeny cells with PDGFRβ-CreER::R26-TdT mice. **B** Representative images showing that PDGFRβ-TdT+ cells (red) are present in the perivascular spaces and meninges of the normal spinal cord. Three repeats are performed independently. The rectangular boxes indicate the enlarged regions in the right images. Scale bars: 250 μm (left), 10 μm (right). **C** Representative images showing PDGFRβ-TdT+ cells surrounding vessels (arrows). Three repeats are performed independently. Arrows indicate the adjacency of TdT (red) and CD31(green). Scale bar: 50 μm. **D** After transection (top) or crush (bottom) spinal cord injury (SCI), PDGFRβ-TdT+ cells (red) migrate into the lesion core. Dashed lines label the lesion regions. Three repeats are performed independently. Scale bars: 250 μm. **E** Quantification of the number of PDGFRβ-TdT+ cells in the lesion core. Data are shown as mean ± SEM. *n* = 3 mice per group. For transection SCI, ns = 0.7900, ****P < 0.0001. For crush SCI, ns = 0.9909, **P = 0.0011 (5 dpi vs. 14 dpi), **P = 0.0015 (5 dpi vs. 1 mpi), by two-way ANOVA with Sidak’s multiple comparisons test. **F** Representative images showing that lesion borders are formed between PDGFRβ-TdT+ cells (red) and GFAP+ astrocytes (green) at 14 dpi. Dashed lines define the lesion regions. Scale bars: 250 μm. **G** Representative images showing that TdT+ cells express Acta2 at 5 dpi after SCI. Arrows indicate the co-localization of TdT (red) with Acta2 (green). Scale bars: 50 μm. **H** PDGFRβ-TdT+ cells (red) in the perivascular space and meninges express Ki67 (green) at 5 dpi. h1–h4, magnified images of the boxed regions. Dashed lines define the lesion regions. Scale bars: 250 μm, 50 μm (h1–h4). **I** Representative confocal images showing the signals for Col1 (green) enriched at the location of TdT+ cells (red) 14 days post SCI. Dashed lines define the lesion regions. Scale bars: 250 μm. Source data are provided as a Source Data file.

To trace PDGFRβ+ cells, we crossed the mouse strain PDGFRβ-CreER with R26-TdT to generate PDGFRβ-CreER::R26-TdT mice (Fig. 1A). In the intact spinal cord, PDGFRβ-TdT+ cells were distributed in the parenchyma and meninges (Fig. 1B, Fig. S1A). In the parenchyma, almost all PDGFRβ+ cells were labeled by TdT. In the meninges, we also found obvious co-localization of TdT and PDGFRβ (Fig. S1A). Vessels were visualized by CD31 and Pdx immunofluorescence, and PDGFRβ-TdT+ cells in the parenchyma were found to be adhered to the endothelial cells, in accordance with the characteristic of perivascular cells (Figs. 1C, S1B, S1E). In addition, we detected that Sox9 was expressed in a subset of TdT+ cells, while Sox10 was not, which was consistent with the previously reported expression of PDGFRβ in a minority of astrocytes but not oligodendrocytes (Figs. S1C, S1D, S1F)^17,21^. These results demonstrate that the perivascular cells and meningeal cells in spinal cords are mainly labeled in PDGFRβ-CreER::R26-TdT mice.

To investigate the dynamic changes of PDGFRβ+ cells post injury, we generated two models, transection SCI (penetrating) and crush SCI (non-penetrating) (Fig. 1D). We compared the densities of PDGFRβ-derived progeny at 5 days post injury (dpi, subacute phase), 14 dpi (time of glia scar formation), and 1 month post injury (mpi, chronic phase). After transection or crush SCI, many PDGFRβ-TdT+ cells were present in the lesion core (Fig. 1D). The densities of PDGFRβ-TdT+ cells at 14 dpi and 1 mpi were relatively higher than those at 5 dpi (Fig. 1D, E). It should be noted that, in the transection SCI model, the density of PDGFRβ-TdT+ cells in the lesion core was higher than that in the crush SCI model (Fig. 1D, E). At 14 dpi, PDGFRβ-TdT+ cells accumulated within the lesion core, thus forming a clear border with GFAP+ astrocytes of the glia scar (Fig. 1F). In addition, PDGFRβ-TdT+ cells were intermingled with CD68+ activated macrophages and CD31+ endothelial cells in the lesion, but no colocalization of TdT and CD68/CD31 was observed (Fig. S1G). We also found very few Sox9+TdT+ cells and no Sox10+TdT+ cells in the lesion core (Fig. S1H). There were some Sox9+TdT+ cells in the surrounding normal area, and the proportion of these cells to TdT+ cells was similar to that of the intact spinal cord (Fig. S1D, H, I). PDGFRβ+ cells expressed high levels of Acta2 at 5 dpi, indicating their conversion into myofibroblasts after SCI (Fig. 1G)^22–24^. Taken together, these observations indicate that PDGFRβ+ cells migrate into the lesion sites and convert into myofibroblasts after both transection and crush SCI. In addition, more PDGFRβ+ cells are recruited to the lesion sites after transection SCI compared to crush SCI.

### Both parenchyma and meningeal PDGFRβ+ cells are activated and contribute to fibrotic scar formation after transection and crush SCI

To examine whether PDGFRβ-TdT+ cells proliferated after SCI, mitotic PDGFRβ-TdT+ cells were identified using the marker Ki67. Both transection and crush SCI induced proliferation of TdT+ cells in the meninges and perivascular space, as demonstrated by the co-expression of TdT and Ki67 (Fig. 1H). At 5 dpi, the percentage of TdT+ cells that were dividing was 49.18 ± 2.86% in the transection SCI model, compared with 43.26 ± 2.24% in the crush SCI model (Fig. S2A). At 14 dpi and 1 mpi, the proportion of proliferating fibroblasts dropped to a very low level (Fig. S2A). We also injected BrdU (50 mg/kg) intraperitoneally after SCI to label proliferating TdT+ cells. Notably, at 14 dpi, the percentage of BrdU+TdT+ cells were about 50% in both transection and crush SCI (Fig. S2E, F). This result suggests that the more PDGFRB cells in fibrotic scar of transection SCI may be attributed to cell migration and early proliferation after injury.

Previous studies have reported that Nestin was expressed in stromal myofibroblasts^25^. Although the precise function of Nestin in myofibroblasts is not fully understood, some studies have suggested that Nestin plays a key role in cell activation or migration^26–28^. Hence, we examined the expression of Nestin in PDGFRβ-TdT+ cells. In both transection and crush SCI lesions, PDGFRβ-TdT+ cells could be classified into two categories based on their spatial location: those located within the parenchyma and those in close proximity to the meninges. It should be noted that both types of cells expressed Nestin (Fig. S2B, C). The percentage of TdT+Nestin+ cells out of all TdT+ cells in the lesion core of transection SCI was higher than that of crush SCI at 5 dpi (Fig. S2D). At 14 dpi and 1 mpi, the proportion of TdT+Nestin+ cells in PDGFRβ-TdT+ cells decreased to a lower level than that at 5 dpi (Fig. S2D). Here, we observed a continuous distribution of Nestin+TdT+ cells or Ki67+TdT+ cells originating from the meninges, as well as Nestin+TdT+ cells or Ki67+TdT+ cells within the parenchyma at 5 days post-injury (Figs. 1H and S2B, C). We speculate that these findings may be related to the activation of PDGFRβ-TdT cells.

Fibrosis is defined by the deposition of ECM components, such as fibronectin (Fn1), collagen I (Col1), and laminin^29^. The distribution of TdT+ cells was in line with that of Col1-exressing cells after both transection and crush SCI at 14 dpi (Fig. 1I). At 5 dpi, TdT+ cells in the parenchyma also expressed Col1, and there was a continuous TdT+Col1+ signal from the meninges to parenchyma in the crush SCI model, which suggested that there might to be two sources of fibrotic scar cells: the perivascular space and meninges (Fig. S2G). In sum, these findings indicate that both parenchymal and meningeal PDGFRβ+ cells are activated and contribute to fibrotic scar formation after either penetrating or non-penetrating SCI.

### Fibroblasts, rather than pericytes/vSMCs, primarily contribute to the formation of fibrotic scars after SCI

PDGFRβ is expressed in pericytes/vSMCs and fibroblasts in the spinal cord. To determine whether the myofibroblasts in fibrotic scar tissue are derived from pericytes/vSMCs or fibroblasts, we used FACS to sort TdT+ cells and performed scRNA-seq of PDGFRβ+ cells from PDGFRβ-CreER::R26-TdT mice after SCI (Fig. S3A). A total of 44,386 cells were obtained from 0 dpi (uninjured spinal cord prior to injury), 5 dpi, and 14 dpi after the exclusion of low-quality cells and potential doublets. A uniform manifold approximation and projection (UMAP) plot and heatmap revealed multiple distinct cell groups, namely fibroblasts, dividing fibroblasts, pericytes/vSMCs, ependymal cells, microglia/macrophages, neutrophils, oligodendrocytes, astrocytes, endothelial cells, and Schwann cells (Fig. S3B–D). Fibroblasts (including dividing fibroblasts) and pericytes/vSMCs expressed TdT at a higher level compared to other cell populations (Fig. S3E). ScRNA-seq showed lower expression levels of TdT in other cell populations, but immunofluorescence assay showed that microglia/macrophages, endothelial cells, and oligodendrocytes did not express TdT (Fig. S1G, H). The expression levels of ECM-related genes were higher in fibroblasts and pericytes/vSMC compared to other cell types (Fig. S3F). Collectively, these findings suggest that fibroblasts and pericytes/vSMC are the most likely candidates for secreting ECM to form fibrotic scars.

**Fig. 2:**
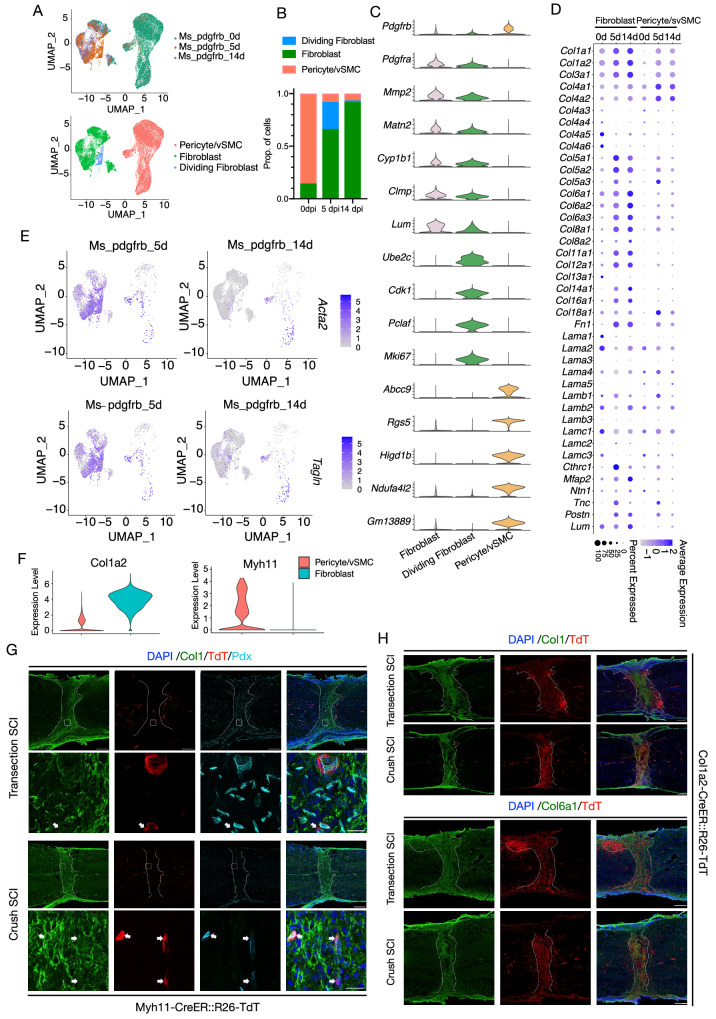
PDGFRβ+ fibroblasts convert into myofibroblasts and express higher levels of ECM proteins than pericytes/vSMCs. **A** UMAP plot of fibroblasts, dividing fibroblasts, and pericytes/vSMCs from scRNA-seq data of spinal cords at 0 dpi (uninjured spinal cord prior to SCI), 5 dpi, and 14 dpi. **B** Bar graph showing the proportion of dividing fibroblasts, fibroblasts, and pericytes/vSMCs relative to the total number of cells at 0 dpi, 5 dpi, and 14 dpi. **C** Violin plots showing the expression patterns of marker genes of fibroblasts, dividing fibroblasts, and pericytes/vSMCs. **D** Dot plot showing the expression patterns of ECM-associated genes in fibroblasts (including dividing fibroblasts) and pericytes/vSMCs. **E** UMAP projection showing the expression patterns of the myofibroblast marker genes Acta2 and Tagln at 5 dpi and 14 dpi. **F** Violin plot showing the different expression of *Col1a2* and *Myh11* in pericytes/vSMCs and fibroblasts. **G** Representative images showing that few TdT+ cells (red) are located in lesion center (Col1-positive cell area, green) and enwrapped by Pdx+ endothelial cells (light blue) at 14 dpi in Myh11-CreER::R26-TdT mice. Three repeats are performed independently. Arrows indicate the adjacency of TdT (red) and Pdx (light blue). Dashed lines define the lesion regions. Scale bar: 250 μm, 25 μm (enlarged view). **H** Representative confocal images showing that the distribution area of TdT+ cells (red) overlapped with that of Col1 (green) and Col6a1 (green) immunofluorescence signals in Col1a2-CreER::R26-TdT mice. Three repeats are performed independently. Dashed lines define the lesion regions. Scale bar: 250 μm. Source data are provided as a Source Data file.

**Fig. 3:**
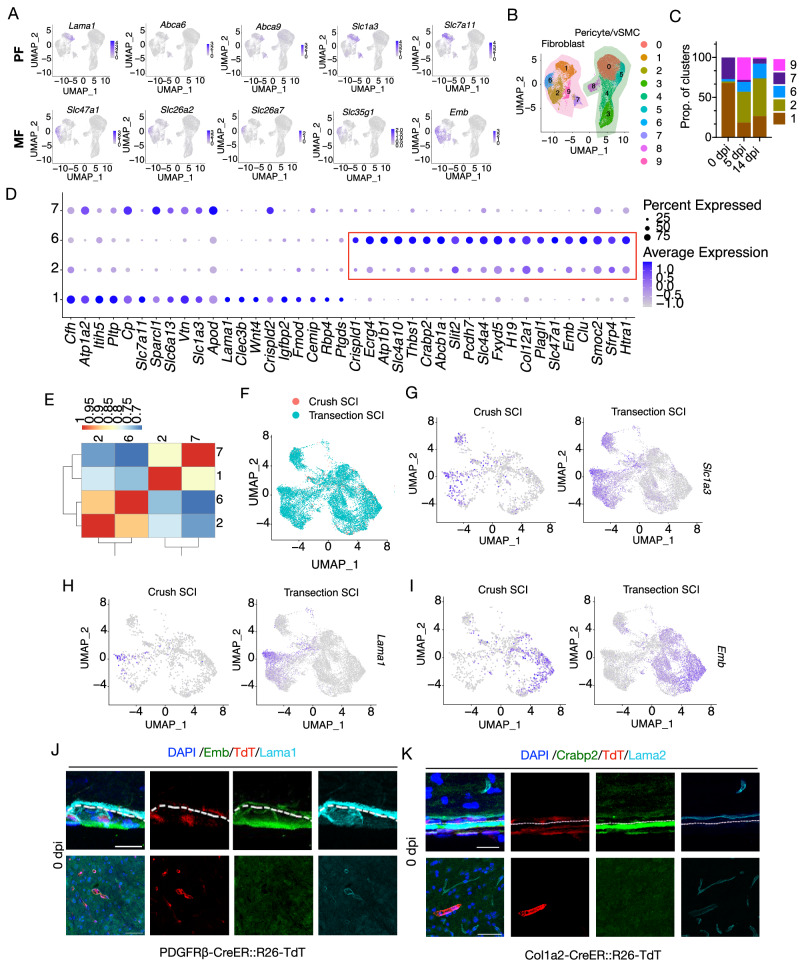
Scar-forming fibroblasts were derived from MFs and PFs with different spatial source. **A** UMAP plots showing the expression patterns of specific markers for perivascular (PF) and meningeal (MF) fibroblasts. **B** UMAP of cluster analysis revealed 10 clusters of fibroblasts (cluster 1, 2, 6, 7), dividing fibroblasts (cluster 9), and pericytes/vSMCs (cluster 0, 3, 4, 5, 8). **C** The proportion of each cluster at 0 dpi, 5 dpi, and 14 dpi. Cluster 9 (dividing fibroblasts) and cluster 2 emerged after injury. **D** Expression patterns of the top 20 DEGs between clusters 1 and 6 in fibroblasts showing the similarity of cluster 2 and 6. **E** Heatmap indicating stronger relationship between cluster 2 and cluster 6. Five-hundred genes with high standard deviation in average expression analysis are selected to do Spearman’s correlation. **F** Integrated UMAP plot of fibroblasts from our transection SCI data and published crush SCI data^31,46^. **G**–**I** UMAP plot showing the expression patterns of PF markers *Lama1* (**G**) and *Slc1a3* (**H**) and MF marker *Emb* (**I**) in crush SCI and transection SCI. **J** The expression patterns of Emb and Lama1 in PDGFRβ-CreER::R26-TdT mice. The immunofluorescence signal of Lama1 is located in the pia that inside of meninges and in the parenchyma, while Emb is mainly expressed in the meninges outside of Lama1. Three repeats are performed independently. Dashed lines distinguish dura/arachnoid and pia mater. Scale bar: 50 μm. **K** The expression patterns of Crabp2 and Lama2 in Col1a2-CreER::R26-TdT mice. The immunofluorescence signal of Lama2 is located in the pia that inside of meninges and in the parenchyma, while Crabp2 is exclusively expressed in the outer dura matter or arachnoid. Three repeats are performed independently. Dashed lines distinguish dura/arachnoid and pia mater. Scale bar: 50 μm. Source data are provided as a Source Data file.

We next clustered fibroblasts (including dividing fibroblasts) and pericytes/vSMCs according to the expression levels of cell-type related genes (Fig. 2A). Class 1 (clusters 1, 2, 6, and 7) was recognized as fibroblasts because the cells in these clusters expressed *PDGFRα, Mmp2, Matn2, Cyp1b1, Clmp*, and *Lum*, while class 2 (cluster 9) was classified as dividing fibroblasts because of the expression of *Ube2c, Cdk1, Pclaf*, and *Mki67*. Class 3 (clusters 0, 3, 4, 5, and 8) cells expressed *Abcc9, Rgs5, Higd1b, Ndufa4l2*, and *Gm13889* and thus were defined as pericytes/vSMCs (Fig. 2A, C and 3B)^17,30,31^. Notably, at 5 dpi and 14 dpi, the ratio of fibroblasts was much higher than that of pericytes/vSMCs, which was different from the lower ratio observed at 0 dpi, suggesting that the proportion of fibroblasts increased substantially after injury (Fig. 2B). The proportion of dividing fibroblasts was obviously lower at 14 dpi compared with that at 5 dpi (Fig. 2A, B), which was in accordance with the results above (Figs. 1H and S2A).

In addition, we examined the expression of ECM-related genes including those encoding collagen, glycoproteins, and proteoglycans in fibroblasts and pericytes/vSMCs at 0 dpi, 5 dpi, and 14 dpi. Fibroblasts expressed higher levels of ECM-related genes than pericytes/vSMCs (Fig. 2D). For example, fibroblasts expressed significantly higher levels of collagen-related genes, such as *Col1a1, Col1a2, Col3a1, Col6a1, Col6a2*, and *Col6a3*, than pericytes/vSMCs, and the expression levels of these genes increased with time after injury. The expression of *Fn1* and *Col12a1*, which play a key role in mediating bridge formation and wound healing after SCI^32,33^, increased persistently after injury, while *Lamb2*^34^ expression was sustained at a low level after SCI. The expression of the axon guidance molecule *Ntn1* remained at a low level both before and after SCI. *Tnc* and *Cthrc1a*, which promote axon regeneration and functional recovery in zebrafish^35,36^, were expressed at low levels at 0 dpi and at higher levels at 5 dpi in the adult mouse spinal cord. *Mfap2* and *Postn*, identified as inhibitors of axon regeneration in the zebrafish SCI model^37^, gradually increased with time after SCI. These data showed that SCI induced increased expression of both genes promoting and inhibiting ECM. At 5 dpi, fibroblasts transiently expressed *Acta2* and *Tagln*, indicating the transformation of fibroblasts into myofibroblasts (Fig. 2E).

We next detected whether PDGFRβ+ cells in the injured area are pericytes/vSMCs or not. In the PDGFRβ-CreER::R26-TdT mice, less than 8% of TdT+ cells were colocalized with Desmin (pericytes/vSMCs marker) after SCI (Fig. S4A, B). *Col1a2* and *Myh11* were specifically expressed in fibroblasts and pericytes/vSMCs, respectively (Fig. 2F). To further exclude the contribution of pericytes/vSMCs to fibrotic scar formation, we used Myh11-CreER::R26-TdT mice to track cell fate of pericytes/vSMCs after SCI. In uninjured spinal cord, Myh11-TdT+ cells expressed marker genes of pericytes or vSMCs, such as CD13, Myh11, Desmin and αSMA (Fig. S4C–G). TdT+ pericytes/vSMCs in the lesion core remained attached to blood vessels (Fig. 2G). It could be concluded that Myh11+ pericytes/vSMCs contribute little to the formation of fibrotic scar. Chondroitin sulfate proteoglycan NG2 (encoded by CSPG4) is a commonly used marker of pericytes/vSMCs and oligodendrocyte progenitor cells^38,39^. We also employed NG2-CreER::R26-TdT mice to label NG2+ pericytes/vSMCs^15,40^. Sox10+ oligodendrocytes, Olig2+ oligodendrocytes and Desmin+ pericytes/vSMCs were labeled in NG2-CreER::R26-TdT mice (Fig. S5A–E). After transection SCI, NG2+ cells were mainly located around GFAP+ glia scars, including Sox10+ oligodendrocytes and Olig2+ oligodendrocytes, but not Desmin+ pericytes/vSMCs (Fig. S5B–E). We also examined the distribution of TdT+ cells at 1 mpi. A few TdT+ cells co-labeled with PDGFRβ at the periphery of the lesion core, but the majority of NG2-derived cells in the lesion core were PDGFRβ negative (Fig. S5F). After excluding the contribution of pericytes/vSMCs, we used Col1a2-CreER::R26-TdT mice to confirm fibroblasts as the primary source of fibrotic scars (Fig. 2H). In the uninjured spinal cord, Col1a2-TdT+ cells are distributed in the meninges or blood vessels of parenchyma (Fig. S6A–C). Col1a2-TdT+ cells exhibited colocalization with Col1 and PDGFRβ (Fig. S6B, C). Col1a2-TdT+ cells accounted for 35.20 ± 1.40% of PDGFRβ+ cells in the parenchyma (Fig. S6D). Quantitative analysis showed that Col1+ cells and PDGFRβ+ cells constituted the majority of TdT+ cells in uninjured spinal cord parenchyma (Fig. S6E). After either transection SCI or crush SCI, TdT+ cells were concentrated in Col1+ injury sites and did not express markers of oligodendrocytes, astrocytes, microglia/macrophage and endothelial cells (Fig. 2H, Fig. S6F). Besides, the injury site was filled with fibroblast specific Col6a1-positive ECM (Fig. 2D, H). We calculated the densities of TdT+ cells in Col1+ area of different transgenic mice. The densities of TdT+ cells were similar in PDGFRβ-CreER::R26-TdT mice and Col1a2-CreER::R26-TdT mice, which were higher than those in NG2-CreER::R26-TdT mice and Myh11-CreER::R26-TdT mice (Fig. S6G). Altogether, these findings indicate that fibrotic scars primarily originate from PDGFRβ+ fibroblasts in the intact spinal cord, but not pericytes/vSMCs.

### Fibroblasts in the central and peripheral regions of fibrotic scar are meningeal Crabp2+/Emb+ fibroblasts (CE-F) and perivascular/pial Lama1+/Lama2+ fibroblasts (LA-F), respectively

To determine whether there are heterogeneous subpopulations of fibroblasts in fibrotic scars, we analyzed fibroblasts in our scRNA-seq data. Cluster 1 and 7 were identified as PFs based on the expression of specific markers: *Abca6, Abca9, Slc1a3*, and *Slc7a11*^41^ (Fig. 3A). Similarly, Cluster 6 was identified as MFs based on expression of *Slc47a1, Emb, Slc26a2, Slc26a7* and *Slc35g1*^41^ (Fig. 3A). To identify the characters of cluster 2 cells increased robustly post injury (Fig. 3B, C), We made parallel comparisons among clusters 1, 2, 6, and 7 and found that cluster 2 was most similar to cluster 6 (Fig. 3E). In addition, cluster 2 expressed more of the top 20 DEGs of cluster 6 than the other clusters, which also revealed the possible meningeal origin (Fig. 3D). Clusters 2 and 6 shared similar expression patterns of ECM-related genes compared with cluster 1 and 7 (Fig. S8A). Therefore, cluster 2 was identified as MFs. Recently, brain leptomeningeal fibroblasts were divided into five subtypes: BFB1-BFB5^42^. BFB1 refers to pial FBs and PFs. BFB2 and BFB3 refers to inner arachnoid FBs. BFB4 refers to arachnoid barrier cells, and BFB5 refers to dural border cells. We found cluster 1 expressed higher levels of *Lama1*, and cluster 7 expressed *Col15a1, Ebf2* and *Apod*, marker genes of BFB1 (pia fibroblasts and perivascular fibroblasts) (Figs. S7A and 3A, D). Cluster 2 and 6 expressed higher levels of maker genes of BFB2 (*Itm2a*), BFB3 (*Sgk1*), BFB4 (*Fn1* and *Cd109*) and BFB5 (*Mgp, Fxyd5* and *Bnc2*) (Fig. S7A). Lama1 has been reported to be expressed by pial cells and migrate into brain parenchyma along arterioles^43^. MFs expressed marker genes for dura fibroblasts (*Fxyd5, Smoc2* and *Slc47a1*) and arachnoid fibroblasts (*Crispdl1*), as well as for dura/arachnoid fibroblast marker gene (*Crabp2*)^44^, we refered clusters 2 and 6 as MFs and clusters 1 and 7 as PFs (Fig. 3D). To better understand the heterogeneity of PFs, cluster 1 and cluster 7 were extracted respectively and were further reclustered into two subtypes (subtype 0 and subtype 1) (Fig. S7B, D). Based on the expression of the marker genes^42^, subtype 0 from previous cluster 1 expressed more genes of BFB1a (parenchyma perivascular fibroblasts) and subtype 1 from previous cluster 1 expressed mixed genes of BFB1a and BFB1b (pia perivascular fibroblasts and pia fibroblasts) (Fig. S7C). Subtype 0 and subtype 1 from previous cluster 7 expressed both marker genes of BFB1a and BFB1b (Fig. S7E). It suggests PFs in our study contain both BFB1a and BFB1b cells, but these PFs can not be completely distinguished as different subsets by the marker genes of BFB1a and BFB1b. It might be related to the difference between brain and spinal cord or the change in  gene expression after SCI. In addition, we also found different expression of *Lama1* and *Lama2* between cluster 1 and cluster 7. Cluster 1 cells highly expressed *Lama1*, while cluster 7 cells highly expressed *Lama2* (Fig. S8A). These data suggest the possible presence of *Lama2*+*/Lama1*− perivascular/pial fibroblasts in spinal cord tissue, which has not been emphasized in previous studies.

In addition, MFs expressed higher levels of *Fn1* and *ColIa1*, while PFs expressed higher levels of *Lama1* and *Col4a1* (Fig. S8A). Two types of fibroblasts in the adult mouse brain have been defined by using single-cell transcriptomics: Lama1+ PFs and fibroblasts specifically expressing *Emb*^17,45^. Consistent with the classification of brain fibroblasts, *Lama1* was also expressed in spinal cord PFs, whereas *Emb* was expressed in MFs (Figs. 3D and S8B). To better understand the heterogeneity of fibroblasts, further cluster analysis of dividing fibroblasts was performed. The overall UMAP shows two major classes consisting of six clusters, which correspond to Lama1+ PFs and Emb+ MFs respectively (Fig. S8C, D). To investigate the similarity of fibrotic scar cells in different SCI models, we performed cluster analysis of fibroblasts after transection SCI and crush SCI^31,46^ and identified similar subclusters of fibroblasts (Fig. 3F). Depending on the expression of *Lama1/Slc1a3* and *Emb*, fibroblasts were also classified into PFs and MFs (Fig. 3G–I).

**Fig. 4:**
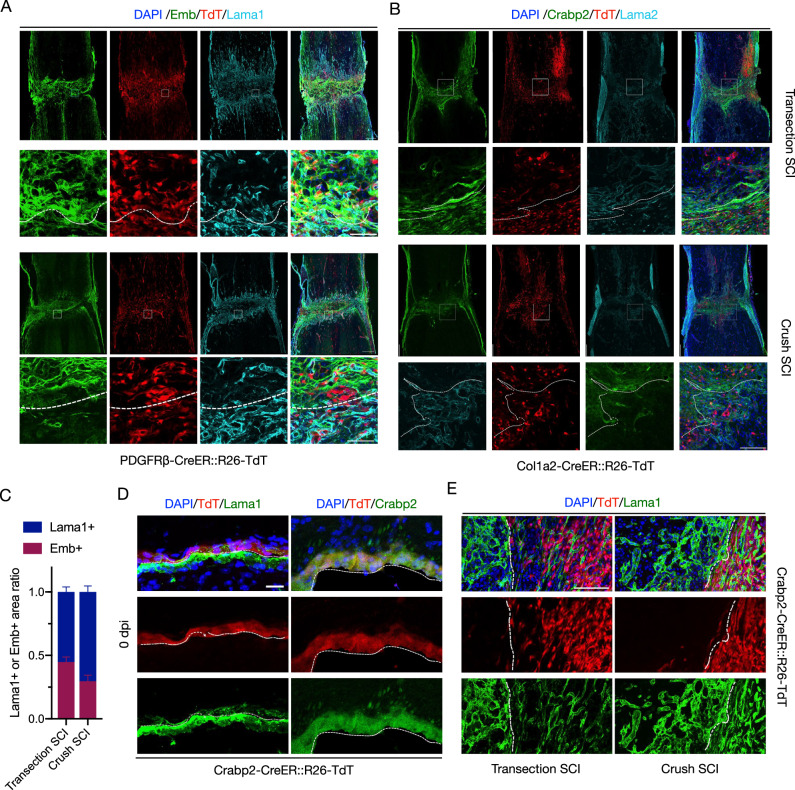
Heterogeneous origin and spatial distribution of fibroblasts in fibrotic scars. **A** Immunostaining for Lama1 (light blue) and Emb (green) at 14 dpi in PDGFRβ-CreER::R26-TdT mice after transection SCI or crush SCI. TdT positive cells are red. Dashed lines distinguish the lesion regions from normal tissues. The rectangular boxes indicate the enlarged regions in the images below. Scale bar: 250 μm, 50 μm (enlarged view). **B** Immunostaining for Lama2 (blue light) and Crabp2 (green) at 14 dpi in Col1a2-CreER::R26-TdT mice after transection SCI or crush SCI. TdT positive cells are red. Dashed lines distinguish the lesion regions from normal tissues. The rectangular boxes indicate the enlarged regions in the images below. Scale bar: 250 μm, 50 μm (enlarged view). **C** Quantification of Lama1+ area index and Emb+ area index in transection SCI and crush SCI. Data are shown as mean ± SEM. *n* = 3 mice per group. **D** Representative images showing the expression pattern of TdT+ cells (red) consistent with that of Crabp2 (green) and locate outside of Lama1+ signals (green) in uninjured Crabp2-CreER::R26-TdT mice. Three repeats are performed independently. Dashed lines distinguish dura/arachnoid from pia mater. Scale bar: 25 μm. **E** There is a low level of distribution of Crabp2-TdT+ cells (red) in Lama1+ (green) exterior of fibrotic scars after either transection SCI or crush SCI. Dashed lines distinguish the lesion regions from normal tissues. Three repeats are performed independently. Scale bar: 100 μm. Source data are provided as a Source Data file.

We then examined the spatial heterogeneity of fibroblasts before and after SCI. In normal PDGFRβ-CreER::R26-TdT mice, we performed immunohistochemical analyses using Lama1 and Emb antibodies. We found that Emb+ signals were localized in outer dura/ arachnoid but Lama1+ signals were mainly localized in the pial and the parenchyma (Fig. 3J). Similarly, in normal Col1a2-CreER::R26-TdT mice, Crabp2+ signals were located outside of Lama2+ signals in the meninges, and Lama2+ signals were also present in the parenchyma (Fig. 3K). In both transgenic mice, TdT+ cells were distributed in both meninges and parenchyma (Fig. 3J, K). After SCI, Emb+/Crabp2+ fibroblasts were localized in the center of lesions and surrounded by Lama1+/Lama2+ fibroblasts (Fig. 4A, B). Moreover, by statistical analysis of Lama1+ and Emb+ areas, it was found that the proportion of Lama1+ cells in crush SCI was relatively higher than that in transection SCI (Fig. 4C).

To specifically track the cell fate of MFs, we then applied CRISPR/Cas9 method to construct Crabp2-CreER::R26-TdT transgenic mice by inserting tamoxifen-inducible Cre recombinase under the control of the endogenous Crabp2 promoter. In the uninjured spinal cord, TdT+ cells consistent with Crabp2 and Emb expression patterns were located outside of Lama1+ signal (Figs. 4D and S9A, B). No colocalization of TdT and CD68 were detected in the lesion core, indicating the Crabp2-TdT+ cells were not the infiltrating microglia/macrophage (Fig. S9C). After SCI, Crabp2-TdT+ cells expressed Crabp2 and Emb and located in the center area of the fibrotic scar, which were surrounded by Lama1+ signals (Figs. 4E and S9D–H). The ratio of Crabp2-TdT+ cells in Emb+ area was significantly higher than that in Lama1+ area (Fig. S9F). In sum, the results demonstrate that the scar-forming fibroblasts can be divided into meningeal Crabp2+/Emb+ fibroblasts (CE-F) and pia/perivascular Lama1+/Lama2+ fibroblasts (LA-F) with special spatial distribution pattern following transection or crush SCI.

### The TGF-β and Wnt signaling pathways are primarily activated in fibroblasts, while the PDGFB/PDGFD signaling pathway is more activated in pericytes/vSMCs

**Fig. 5:**
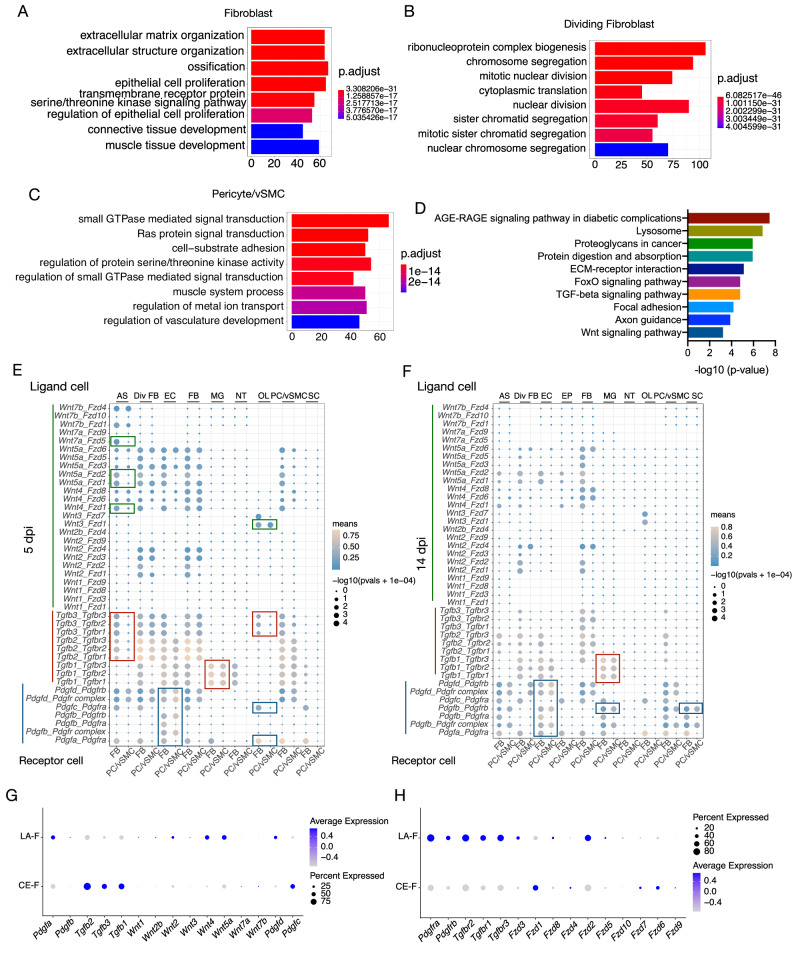
The heterogeneous activation of scar-related TGF-β, Wnt and PDGF signaling pathways in pericytes/vSMCs and fibroblasts. **A**–**C** GO enrichment analysis of differentially expressed genes (DEGs) among fibroblasts, dividing fibroblasts, and pericytes/vSMCs in the injured spinal cord. *y*-Axis shows the top 10 GO biological process terms, and the *x*-axis shows −log10 (FDR-corrected *P* values). *P*-values (two-tailed *p*-value) were calculated by hypergeometric distribution method and FDR were used to estimate an adjusted *p*-value (*q*value). **D** KEGG pathway enrichment analysis of DEGs in fibroblasts compared with dividing fibroblasts and pericytes/vSMCs showing the enrichment of the TGF-β and Wnt signaling pathways in fibroblasts at 5 dpi and 14 dpi. *y*-Axis shows signaling pathway terms, and *x*-axis showing −log10 (FDR-corrected *P* values). *P*-values (two-tailed *p*-value) were calculated by hypergeometric distribution method and FDR were used to estimate an adjusted *p*-value (qvalue). **E**, **F** The algorithm of cellphoneDB was used to do cell–cell interactions analysis. Ligand-receptor analysis of TGF-β, Wnt, and PDGF signaling pathways to assess the interactions of fibroblasts and pericytes/vSMCs with other cell types at 5 dpi (**E**) and 14 dpi (**F**). Left axis shows the specific ligand–receptor pairs. The size of dots indicates the −log10 (*P* value) (two-tailed *p*-value), while color of dots represents the interaction score. AS astrocyte, Div FB dividing fibroblast, EC endothelial cell, FB fibroblast, MG microglia/macrophage, NT neutrophil, OL oligodendrocyte, PC/vSMC pericyte/vSMC, SC Schwann cell. The rectangular boxes in the images highlight difference between FB and PC/vSMC. **G**, **H** Dot plots showing the expression of ligand genes (**G**) and receptor genes (**H**) associated with TGF-β, Wnt, and PDGF signaling pathways in CE-F and LA-F.

We next analyzed the fibrosis-related signaling pathways in pericytes/vSMCs and fibroblasts. After SCI, enriched gene ontology (GO) terms for fibroblasts were ECM organization and extracellular structure organization, while those for dividing fibroblasts were related to nuclear division (Fig. 5A, B). In contrast, the enriched GO terms for pericytes/vSMCs were related to small GTPase-mediated signal transduction and Ras protein signal transduction^47^ and vasculature development (Fig. 5C). The TGF-β, Wnt, and PDGF signaling pathways are involved in CNS fibrotic scar formation^14,18,48^. Here, Kyoto Encyclopedia of Genes and Genomes (KEGG) enrichment analysis also showed that the enriched pathways for fibroblasts at 5 and 14 dpi were TGF-β and Wnt signaling pathways (Fig. 5D).

Next, we employed CellPhoneDB to calculate the interaction scores of ligand-receptor pairs (ISLRPs) for TGF-β, Wnt, and PDGF between multiple cell types and pericytes/fibroblasts based on the average expression levels. The ISLRPs of TGF-β and Wnt were higher at 5 dpi than 14 dpi, but the ISLRPs of PDGF were higher at 14 dpi than 5 dpi, suggesting that these fibrotic pathways had different roles at early and late stages after SCI (Fig. 5E, F). The ISLRPs of TGF-β and Wnt between fibroblasts and other cell types was higher than those between pericytes/vSMCs and other cell types at both 5 dpi and 14 dpi, indicating the important role of fibroblasts during fibrosis. The ISLRPs of PDGFB and PDGFD between pericytes/vSMCs and endothelial cells (ECs) were higher than those between fibroblasts and ECs (Fig. 5E, F), indicating the important role on angiogenesis of pericytes/vSMCs.

We also found that the ISLRPs of *Wnt7a-Fzd5, Wnt5a-Fzd2, Wnt5a-Fzd1, Wnt4-Fzd1, Tgfb3-Tgbfrs*, and *Tgbf2-Tgbfrs* between astrocytes and fibroblasts were higher than those between astrocytes and pericytes/vSMCs at 5 dpi, indicating that Wnts and TGFs contributed to early interaction between astrocytes and fibroblasts (Fig. 5E, F). In addition, the ISLRPs of *Wnt3-Fzd7, Tgfb3-Tgfbrs, Pdgfc-Pdgfra*, and *Pdgfa-Pdgfra* between oligodendrocytes and fibroblasts were higher than those between oligodendrocytes and pericytes/vSMCs (Fig. 5E, F). The ISLRPs of *Tgfb1-Tgfbrs* between microglia/macrophages and fibroblasts were higher than those between microglia/macrophages and pericytes/vSMCs at 5 dpi and 14 dpi, but the *Pdgfb-Pdgfrb* interaction between pericytes/vSMCs and microglia/macrophages was stronger at 14 dpi (Fig. 5E, F). This suggested that there were different interactions between microglia/macrophages and fibroblasts and pericytes/vSMCs at the early and late stages after SCI. We also detected interactions between Schwann cells (SCs) and pericytes/vSMCs and between Schwann cells and fibroblasts through *Pdgfb-Pdgfrb* at 14 dpi, and the ISLRP of *Pdgfb-Pdgfrb* between pericytes/vSMCs and SC was higher than that between fibroblasts and SCs (Fig. 5E, F). Collectively, our data suggested that the TGF-β and Wnt signaling pathways are primarily activated in fibroblasts and participate in fibrosis, while the PDGFB/PDGFD signaling pathway is more highly activated in pericytes/vSMCs.

We also examined the activation of TGF-β, Wnt and PDGF signaling pathways in other cell types (Fig. S10). Overall, the expression level of ligand and receptor genes were higher in fibroblasts compared with other cell types. In addition, TGF-β signaling and Wnt signaling were activated in Schwann cells and endothelial cells, and Wnt signaling was also activated in astrocytes. It suggests the possible contribution of Schwann cells, endothelial cells and astrocytes for fibrotic scar.

Furthermore, we analyzed expression patterns of ligand and receptor genes in CE-F and LA-F (Fig. 5G, H). *Tgfb1, Tgfb2* and *Tgfb3* were higher expressed in CE-F than in LA-F, while corresponding receptor genes were higher expressed in LA-F. The expression levels of receptor genes of PDGF signaling pathway in LA-F were higher than that in CE-F. LA-F expressed higher levels of Wnt pathway ligand genes *Fzd2* than CE-F, while CE-F expressed higher level of *Fzd1*. It suggests TGF-β, PDGF and Wnt pathway are differently activated in LA-F and CE-F.

### The heterogeneity of fibroblasts in fibrotic scars after SCI is conserved between mice and monkeys

**Fig. 6:**
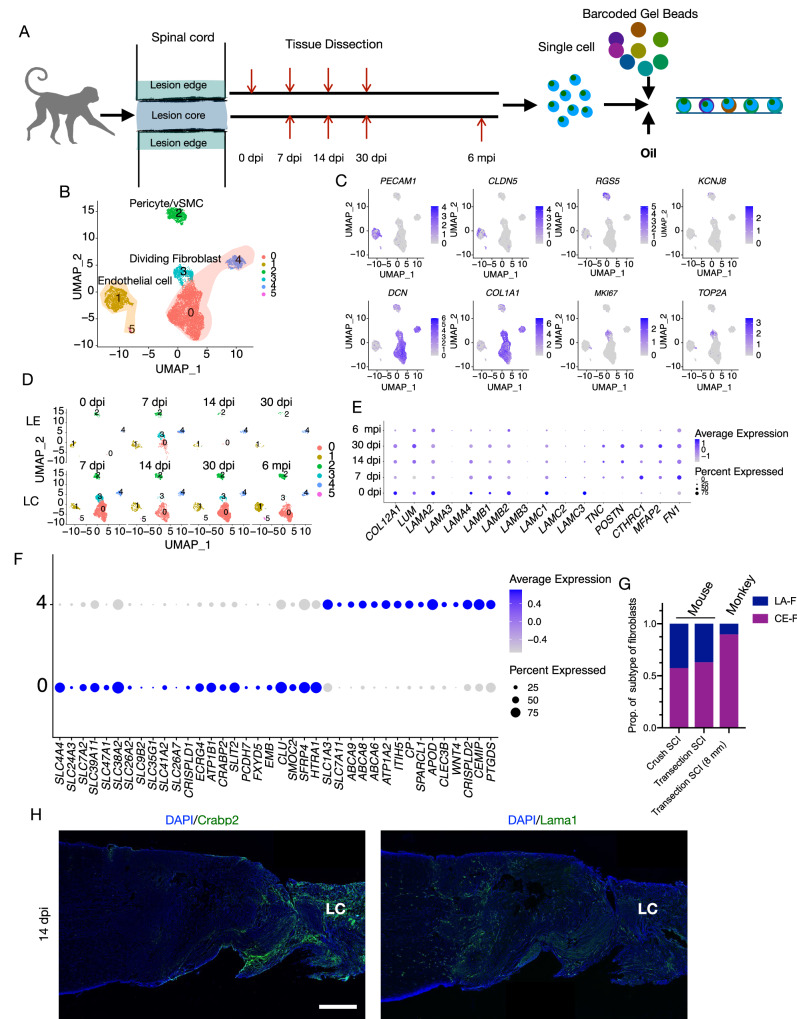
Monkey PFs and MFs participate in fibrotic scar formation after transection SCI with a similar pattern to that observed in mice. **A** Schematic diagram depicting the dissection of spinal cord tissues in the lesion edge and lesion core at 0 dpi, 7 dpi, 14 dpi, 30 dpi, and 6 mpi. Single-cell suspensions are used for subsequent 10× Genomics analysis. 0 dpi refers to uninjured spinal cord. **B** UMAP plot of endothelial cells (clusters 1 and 5), pericytes/vSMCs (cluster 2), dividing fibroblasts (cluster 3), and fibroblasts (clusters 0 and 4) in uninjured spinal cord and injured spinal cord. **C** UMAP plot of expression patterns of canonical marker genes to identify each cell type. **D** UMAP plot of cells in the lesion edge (LE) and lesion core (LC) spilt by each time point. **E** The expression patterns of ECM genes in fibroblasts at different time points. **F** Expression patterns of representative DEGs in fibroblasts that distinguish monkey CE-F and LA-F. **G** The ratio of CE-F and LA-F in mouse and monkey SCI models. **H** Representative images showing the expression pattern of Crabp2 (green in left image) or Lama1 (green in right image) at 14 dpi. LC indicate lesion core. Scale bar: 1 mm. Source data are provided as a Source Data file.

To further verify the results in mice, we established a transection SCI model of *Macaca mulatta*, and dissected the lesion edge and lesion core tissues at different time points after SCI for 10× Genomics sequencing and analysis (Fig. 6A). Canonical marker genes for endothelial cells (*PECAM1* and *CLDN5*), pericytes/vSMCs (*RGS5* and *KCNJ8*), fibroblasts (*DCN* and *COL1A1*), and dividing fibroblasts (*MKI67* and *TOP2A*) were identified according to expression pattern and used to perform cluster analysis (Fig. 6B, C). Dividing fibroblasts were observed at 7 dpi and subsequently decreased in abundance (Fig. 6D). The expression levels of ECM-related genes were altered post injury (Fig. 6E). *TNC* and *CTHRC1A*, which promote ECM deposition, initially increased and then decreased post injury (Fig. 6E). *FN1* expression was higher post injury, while the expression levels of *LAMB2* and other laminin subunit genes were lower after SCI (Fig. 6E). These changes in these genes promoting ECM deposition were similar to those observed in mice. Notably, *COL12A1* expression persistently decreased after SCI in monkeys (Fig. 6E). The expression of genes inhibiting ECM deposition *POSTN* and *MFAP2*, remained at a high level until 30 dpi, then dropped to pre-injury levels (Fig. 6E). Collectively, these observations indicate that fibroblasts express both genes promoting and inhibiting ECM deposition after monkey SCI.

It should be noted that, to distinguish between MFs and PFs in monkey spinal cord, we removed the meninges of normal spinal cord prior to scRNA-seq. Only one small cluster of fibroblasts, cluster 4, was observed at 0 dpi, suggesting that cluster 4 contains PFs but not MFs. Cluster 4 cells in the lesion core after SCI were also identified as PFs, while cluster 0 in lesion edge were MFs (Fig. 6D). At 14 dpi and 30 dpi, most of the fibroblasts at the lesion edge were PFs (Fig. 6D), which indicating that PFs at the edge and MFs in the center of the fibrotic scar (Fig. 6D). This conclusion was also confirmed by the expression patterns of the DEGs obtained from mouse fibroblasts in macaque fibroblasts (Fig. 6F). Consistent with observations in mice, monkey MFs expressed higher levels of *CRABP2, EMB, COL1A1, COL1A2*, and *FN1*, while PFs expressed collagen IV and laminin subunit-related gene *LAMA2* (Fig. 6F and Fig. S11), which can be also denoted as CE-F and LA-F, respectively. We also found more CE-F were recruited to the lesion core in monkeys than in mice, suggesting larger injury area leading to more MFs infiltrated (Fig. 6G). Immunostaining indicated that CRABP2+ signals was located in the center and LAMA1+ signals at the edge of the fibrotic scar, in line with the phenomenon observed in mice (Fig. 6H). In sum, heterogeneity of fibroblasts in fibrotic scar tissue after SCI is observed in both mice and monkeys.

### LA-F participate in lipid transport function after spinal cord injury

**Fig. 7:**
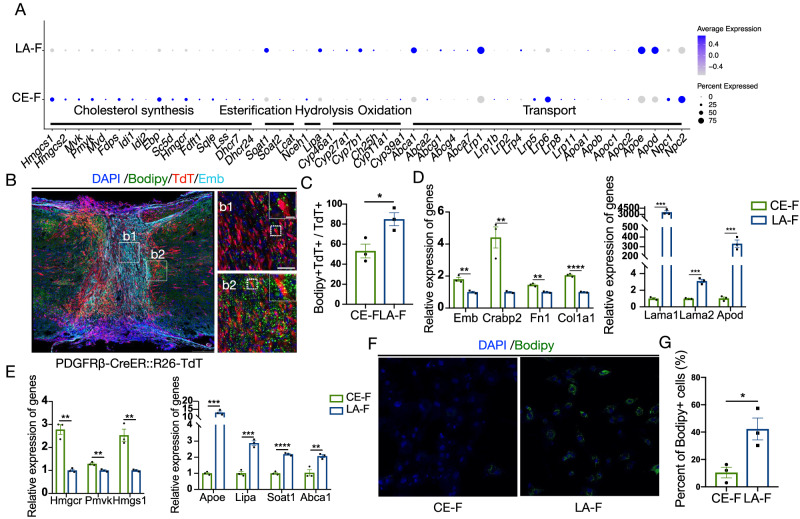
LA-F participate to lipid transport while CE-F possess the robust ability to synthesize cholesterol. **A** Dot plot showing the expression pattern of cholesterol metabolism related genes in mouse CE-F and LA-F. **B** Representative images showing the distribution of Bodipy+ lipid droplets (green) in PDFGRβ-CreER:R26-TdT mice at 14 dpi after transection SCI. Emb is light blue and TdT is red. The rectangular boxes in the left image indicate the enlarged regions in the right images. Scale bars: 250 μm, 50 μm (b1 and b2), 10 μm (enlarged view of b1 and b2). **C** The percentage of Bodipy+TdT+ cells in TdT+ CE-F and LA-F. Data are shown as mean ± SEM. *n* = 3 mice per group. *P = 0.00286 by two-sided, unpaired Student’s *t*-test. **D** The relative expression of marker genes of CE-F and LA-F in cultured cells in vitro by RT-qPCR. Data are shown as mean ± SEM. *n* = 3 samples per group. **P = 0.004197 (*Emb*), **P = 0.006730 (*Crabp2*), **P = 0.001542 (*Fn1*), ****P = 0.000045 (*Col1a1*), ***P = 0.000569 (*Lama1*), ***P = 0.000520 (*Lama2*), ***P = 0.000882 (*Apod*) by two-sided, unpaired Student’s *t*-test. **E** The relative expression of cholesterol metabolism related genes in cultured CE-F and LA-F in vitro by RT-qPCR. Data are shown as mean ± SEM. *n* = 3 samples per group. **P = 0.001213 (*Hmgcr*), **P = 0.005505 (*Pmvk*), **P = 0.004288 (*Hmgcs1*), ***P = 0.000185 (*Apoe*), ***P = 0.000468 (*Lipa*), ****P = 0.000096 (*Soat1*), **P = 0.008737 (*Abca1*) by two-sided, unpaired Student’s *t*-test. **F** LA-F phagocytose myelin sheaths and forms lipid droplets (green) in vitro. Scale bar: 100 μm. **G** The percentage of CE-F and LA-F that containing Bodipy+ lipid droplets in vitro. Data are shown as mean ± SEM. *n* = 3 samples per group. *P = 0.00231 by two-sided, unpaired Student’s *t*-test. Source data are provided as a Source Data file.

SCI often leads to a notable accumulation of lipids derived from myelin debris that can be observed in the lesion, extending well beyond the epicenter^49,50^. We analyzed the function of fibroblasts on lipid metabolism. CE-F highly expressed genes related to cholesterol synthesis (*Hmgcr, Pmvk, Hmgcs1*), while LA-F highly expressed genes related to cholesterol transport (*Apod, Apoe, Abca1* and *Lrp1*) (Fig. 7A). After SCI, lipid droplets (LDs) were found. In PDGFRβ-CreER::R26-TdT mice, the proportion of LA-F containing Bodipy+ LDs was significantly higher than that of CE-F (Fig. 7B, C). We also isolated and cultured rodent CE-F from dura/arachnoid and LA-F from pia in vitro. Consistent with the results described above, CE-F highly expressed *Emb, Crabp2, Fn1* and *Col1a1*, and LA-F highly expressed *Lama1, Lama2* and *Apod* (Fig. 7D). Additionally, in vitro experiments confirmed that CE-F highly expressed genes related to cholesterol synthesis (*Hmgcr, Pmvk, Hmgcs1*), while LA-F highly expressed genes related to cholesterol transport (*Apoe* and *Abca1*), esterification (*Soat1*) and hydrolysis (*Lipa*) (Fig. 7E). When incubating fibroblasts with myelin sheaths, the proportion of LA-F that phagocytosed myelin and then formed LDs was also higher than CE-F, corresponding to in vivo results (Fig. 7F, G). Together, it suggests LA-F has lipid transport function both in vitro and in vivo.

### LA-F play a significant role in promoting vascular formation compared to CE-F

**Fig. 8:**
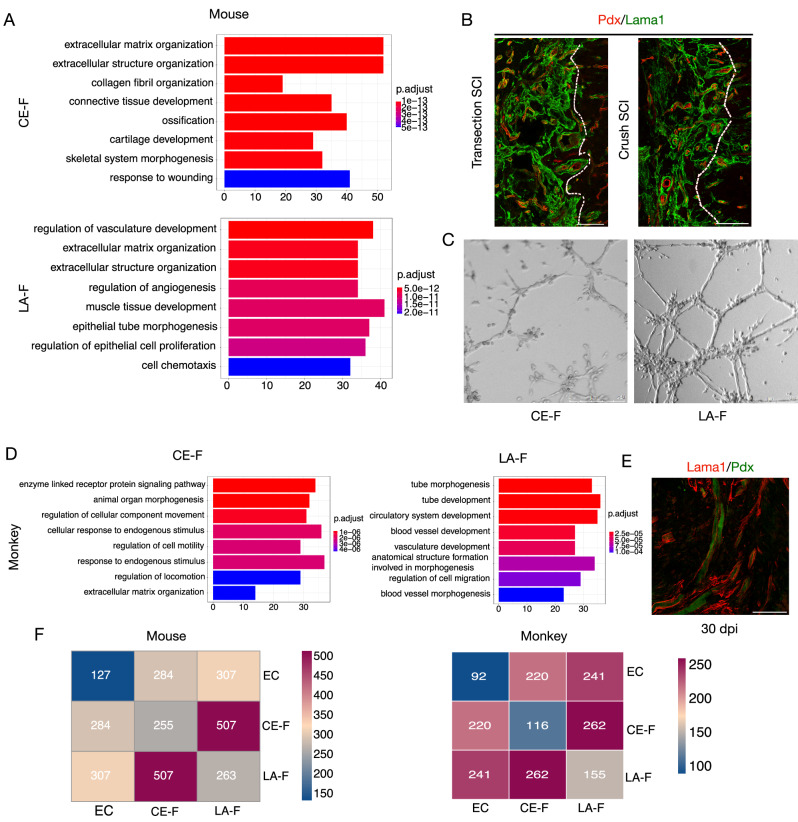
LA-F contribute to blood vessel formation. **A** Biological process terms identified from GO enrichment analysis of the top DEGs between mouse CE-F and LA-F in the injured spinal cord. *P*-values (two-tailed *p*-value) were calculated by hypergeometric distribution method and FDR were used to estimate an adjusted *p*-value (qvalue). **B** Representative confocal images showing that Pdx+ blood vessels (red) are surrounded by Lama1+ fibroblasts (green) in the lesion core. Three repeats are performed independently. Dashed lines distinguish the lesion regions from normal tissues. Scale bars: 100 μm. **C** Endothelial cell tube formation after being co-culture with CE-F or LA-F in vitro. Three repeats are performed independently. Scale bars: 250 μm. **D** Biological process terms identified by GO enrichment analysis of DEGs between CE-F and LA-F after SCI in monkeys. *P*-values (two-tailed *p*-value) were calculated by hypergeometric distribution method and FDR were used to estimate a adjusted *p*-value (qvalue). **E** Representative confocal image showing the expression pattern of endothelial cells (Pdx+, green) and perivascular fibroblasts (Lama1+, red) in monkeys at 30 dpi. Three repeats are performed independently. Scale bar: 100 μm. **F** Heat maps derived from ligand–receptor interaction counts depicting the interactions between CE-F, LA-F, and EC (endothelial cell) of mice (5 dpi) and monkeys (7 dpi).

We performed GO enrichment analysis of the DEGs between mouse CE-F and LA-F. Both CE-F and LA-F shared enriched biological process terms related to ECM organization, whereas the top enriched biological processes for LA-F pertained to vascular development and angiogenesis (Fig. 8A). Immunostaining for Lama1 and Pdx in the transection and crush SCI models showed that Pdx+ signals were surrounded by Lama1+ signals, suggesting that LA-F supported the functions of vessels in the lesion core (Fig. 8B). To further investigate the role of CE-F and LA-F on angiogenesis, rodent fibroblasts and endothelial cells were co-cultured using transwell. Compared to CE-F, LA-F promoted the formation of blood vessels in vitro (Fig. 8C). In monkeys, we also performed GO enrichment analysis of the DEGs between LA-F and CE-F in the lesion core and found that LA-F were associated with blood vessel and vasculature development (Fig. 8D). The Pdx+ endothelial cells were surrounded by Lama1+ LA-F, which was consistent with the observations in mice (Fig. 8E). The interaction between LA-F and endothelial cells was stronger than that between CE-F and endothelial cells (Fig. 8F). Overall, these results suggest that perivascular/pial LA-F contributes to vessel formation after SCI.

## Discussion

**Fig. 9:**
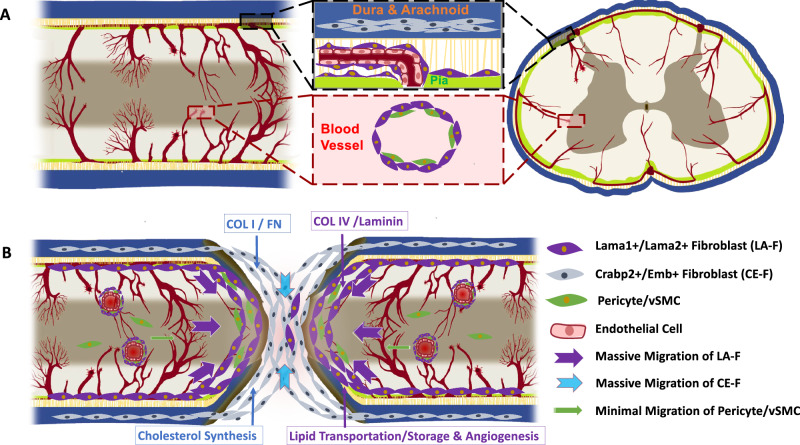
Schematic summarization of heterogeneous contribution of PDGFRβ cells and their progeny in fibrotic scar after transection and crush SCI. **A** In the intact spinal cord, there are two types of fibroblasts, meningeal CE-F in dura and arachnoid, and LA-F in the pia and parenchyma. Pericytes/vSMCs are mainly located around blood vessels in spinal cord parenchyma. **B** After SCI, CE-F and LA-F migrate into the lesion core and generate ECM. CE-F express higher levels of Col1 and Fibronectin, and LA-F express higher levels of Col IV and Laminin. CE-F are active in cholesterol synthesis, and LA-F are involved in lipid transportation/storage and angiogenesis. CE-F and LA-F exhibit a specific spatial distribution within the fibrotic scar region, where LA-F are found on the lateral side of CE-F. Pericytes/vSMCs contribute little to the fibrotic scar formation.

It has been reported that fibrotic scars originate from both MFs and PFs in a dorsal hemisection model where the dura is torn^15^. In contrast, after contusive SCI, where the dura remains intact, the fibrotic scar originates from PFs instead of MFs^15^. Our results demonstrate a dual source of PDGFRβ+ cells in the lesion core after either penetrating or non-penetrating SCI, regardless of whether the dura is intact. Crush (penetrating) and transection (non-penetrating) SCI give rise to similar patterns of PDGFRβ-derived cells, despite the relatively lower density of PDGFRβ+ cells in crush SCI compared with transection SCI. We found the minimal contribution of pericytes/vSMCs to fibrotic scar formation. There were differences in the distribution, cellular characteristics, and functions of MFs and PFs in fibrotic scars. Meningeal CE-F were located in the center of the fibrotic scar, and perivascular/pial LA-F in the edge of the fibrotic scar. CE-F expressed higher levels of genes related with cholesterol synthesis, and LA-F was involved in cholesterol transport and angiogenesis. CE-F and LA-F also differed in the expression of ECM related genes (Fig. 9).

A series of studies have used Glast-CreER mice in combination with reporter mice to trace the fate of Glast+ cells following SCI and found that a small proportion of PDGFRβ+ cells lining capillaries and meninges, termed type A pericytes, contribute to stromal fibroblasts after either penetrating or non-penetrating SCI^7,8,16^ and traumatic brain injury, ischemic stroke, multiple sclerosis, and glioblastoma^16^. However, a recent study demonstrated that the fibrotic scar is derived predominantly from meningeal fibroblasts and perivascular fibroblasts after EAE using Col1a2-CreER mice crossed with reporter mice^2^. Recently, scRNA-seq has been performed to investigate the molecular definitions of blood vascular and vessel-associated cells in the adult mouse brain^17^. This study revealed that Glast (Slc1a3) is expressed in astrocytes, pericytes, and fibroblasts^14,17^. We utilized Myh11-CreER::R26-TdT and NG2-CreER::R26-TdT to trace pericytes/vSMCs. Our results showed pericytes/vSMCs were minimally involved in the formation of fibrotic scar tissue.

To elucidate the identity of scar-forming fibroblasts after SCI, we performed scRNA-seq of PDGFRβ+ cells and lineage tracing with Col1a2-CreER mice and Crabp2-CreER mice. Our data revealed that the vast majority of scar cells after SCI are fibroblasts. Furthermore, we also identified two types of fibroblasts in the spinal cords of adult mice, PFs and MFs, both of which contribute to fibrotic scar formation with different spatial location. Of note, Glast+ cells, previously thought of as type A pericytes^7,8,16^, are actually PFs. However, it is worth to note that pericytes are a heterogeneous cell population^51–53^. It cannot be completely ruled out that a subset of pericytes are transformed into myofibroblasts after spinal cord injury and lose their pericyte properties. Meanwhile, Myh11+ and NG+ pericytes do not represent the whole spinal cord pericytes. More in-depth studies are needed to investigate heterogeneity of pericytes in the central nervous system.

It has been reported that macrophage depletion leads to attenuation of fibrosis after SCI^54^, but the crosstalk between macrophages and fibroblasts has remained unclear. Our data suggest that microglia/macrophages secrete TGFB ligands that activate the TGF-β signaling pathway in fibroblasts and thereby promote fibrosis. High TGFB/TGFBR interaction scores were also present in pericytes/vSMCs at 5 dpi. Previous studies have indicated that TGFB secreted by endothelial cells binds to TGFBR2 in pericytes and activates downstream signaling pathways, thereby inhibiting pericyte proliferation and inducing the expression of contractile protein and ECM proteins^55^. The interaction score of PDGFB-PDGFRB was high in both endothelial cells/fibroblasts and endothelial cells/pericytes. PDGFB secreted by the endothelial cells binds to PDGFRβ on pericytes and initiates a variety of signal transduction pathways to regulate pericyte proliferation, migration, and recruitment to the vascular wall^56,57^. In pericyte-specific PDGFRβ signaling deficient mice, pericyte loss leads to decreased brain microcirculation and Blood-brain barrier (BBB) breakdown, ultimately resulting in neuroinflammation and neurodegenerative changes^58^.

Previous studies have shown that complete ablation of fibrotic scars is not conducive to damage repair, but partial reduction of scars promotes axon regeneration and functional recovery^7–13^. In this study, we found the heterogeneous functions of meningeal-derived CE-F and perivascular/pial LA-F after SCI on lipid synthesis and lipid transport. It suggests that specific ablation of subsets of fibroblasts may differently affect the injured microenvironment and functional recovery after SCI.

## Methods

### Transgenic mice and rhesus macaques

PDGFRβ-CreER::R26-TdT mice were generated by crossing PDGFRβ-CreER mice^59^ (obtained from the Jackson Laboratory, Jax stock no. 030201) with the Rosa26-tdtomato cre reporter line (Jax stock no: 007909). CreER and R26-TdT mice resulting from this hybridization were either homozygous or heterozygous. Similarly, NG2-CreER mice^60^ (obtained from the Jackson Laboratory, Jax stock no. 008538), Col1a2-CreER mice^61^ (obtained from the Jackson Laboratory, Jax stock no. 029567), and Myh11-CreER mice^62^ (obtained from the Jackson Laboratory, Jax stock no. 019079) were also crossed with the Rosa26-tdtomato cre reporter line to generate NG2-CreER::R26-TdT mice, Col1a2-CreER::R26-TdT mice, and Myh11-CreER::R26-TdT mice, which are heterozygous for CreER and homozygous or heterozygous for R26-TdT. We developed Crabp2-CreER mice by using CRISPR/Cas9 to insert CreER fragments before Crabp2’s stop codon.

All mice were more than 8 weeks old at the beginning of the experiments and included both males and females. Mice were housed in a barrier system with a 12 h/12 h light/dark cycle, where temperature and humidity were controlled within a range suitable for mice survival. Water and food were sterilized and freely accessible to mice. Ten female rhesus macaques (*Macaca mulatta*) aged 4–7 years old were housed in Beijing Institute of Xieerxin Biology Resource. Experimental procedures were performed according to *Guide for the Care and Use of Laboratory Animals* which were formulated by the National Institutes of Health (USA) and approved by the Animal Care and Use Committee of the Institute of Genetics and Developmental Biology, Chinese Academy of Sciences, and Use Committee of Beijing Institute of Xieerxin Biology Resource. The approved animal protocol number of monkeys is no. 20191017, and the approved animal protocol number of mice is AP2019003 and AP2023031.

### Tamoxifen treatment

One gram of tamoxifen (T5648, Sigma) was dissolved in 50 mL corn oil (C116023, Aladdin) to make up a solution with a concentration of 20 mg/mL. Adult mice received intraperitoneal injection of tamoxifen solution at 0.1 mg/g for 5 consecutive days.

### BrdU injection

To label proliferating cells, mice were intraperitoneally administered with BrdU (50 mg/kg of body weight) once a day for 13 consecutive days from 1 dpi to 13 dpi. Mice were sacrificed at 14 dpi.

### Surgical procedures

Mice were anesthetized by intraperitoneal injection of sodium pentobarbital. Back fur was removed with a razor blade, and skin was washed with povidone iodine solution. Then an incision in the skin and a midline incision above the thoracic spine were made. A laminectomy was performed to expose the T10 vertebra. For transection SCI (penetrating), the exposed spinal cord was cut fully along the coronal plane with microsurgical scissors. For crush SCI (non-penetrating), the exposed spinal cords were completely crushed from two sides for 5 s with forceps, with dura intact. After the introduction of both types of SCI, the muscle layers and the skin were sutured successively. After completing the surgery, mice were placed in a heated incubator until fully awake from anesthesia. During the experiment, the bladders of mice who underwent surgery were artificially emptied twice a day.

For Rhesus macaques, ketamine (1 mg/kg) was used for intramural anesthesia, and 1.5%–2.5% isoflurane was used for anesthesia. The physiological indexes of monkeys were monitored during the operation, namely oxygen saturation, cardiac rate, and respiratory frequency, with venous transfusion of saline solution. The SCI procedures were similar to those used for mice. Briefly, an incision was made in the skin and muscle, the T9 lamina was removed, and a cut was made through the spinal membrane, thus exposing the spinal cord. Eight millimeters of spinal cord tissue was removed to ensure complete transection of the spinal cord. After successful hemostasis, the dura was sutured, followed by the muscle and skin.

### Tissue dissection and immunofluorescence analysis

Mice were euthanized by intraperitoneal injection of excess sodium pentobarbital and then transcardially perfused with phosphate-buffered saline (PBS) solution for removing all the blood, followed by perfusion with 4% paraformaldehyde (PFA). Spinal cords were dissected and immersed in 4% PFA overnight at 4 °C. PFA-fixed tissues were then incubated with 30% sucrose overnight at 4 °C, embedded in OCT (4583, SAKURA), and stored at −80 °C. Coronal and horizontal sections were cut at 12–15 μm thickness using a cryostat (Leica), stuck to slides, and stored at −80 °C.

Sections were incubated in blocking solution (10% donkey serum and 0.3% Triton-X100) for 1 h at room temperature and then incubated in primary antibody solution diluted with 10% donkey serum (36116ES10, YEASEN) overnight at 4 °C. The primary antibodies used in the experiments are as follows. CD31 (1:200, rat, BD Biosciences), Podocalyxin (Pdx, 1:500, goat, R&D Systems), Vimentin (1:500, rabbit, Abcam), PDGFRβ (1:250, rabbit, Abcam), Crabp2 (1:200, rabbit, Proteintech), Lama2 (1:200, rat, Sigma), Acta2 (1:200, rabbit, Abcam), Col6a1 (1:200, rabbit, Proteintech), Sox9 (1:500, goat, R&D Systems), Sox10 (1:500, rabbit, Abcam), BrdU (1:500, rat, Abcam), Desmin (1:500, rabbit, Abcam), GFAP (1:500, rabbit, Abcam; 1:500, chicken, Abcam), Ki67 (1:500, rabbit, Abcam), Nestin (1:200, chicken, Abcam), Col1 (Cola1a1 and Col1a2, 1:250, rabbit, Abcam), Fibronectin (1:250, rabbit, Abcam), NG2 (1:200, rabbit, Sigma), CD68 (1:500, rabbit, Abcam), Laminin (Lama1) (1:500, rabbit, Sigma), Emb (1:100, rat, Invitrogen), Olig2 (1:500, rabbit, Abcam), CD13 (1:500, rat, Abcam) and Myh11 (1:100, mouse, Santa Cruz). Following incubation in primary antibodies, slides were washed three times with PBS and incubated with secondary antibodies for 1 h at room temperature. For BrdU, tissue sections were incubated with 2 N HCl 37 °C for 25 min, then switched to 0.1 M sodium borate to neutralize for 10 minutes (pH 8.0). The secondary antibodies used (all diluted at 1:500 in PBS) are as follows: goat anti-chicken Alexa Fluor 488 (Thermo Fisher, A11039), donkey anti-rabbit Alexa Fluor 488 (Thermo Fisher, A21206), donkey anti-rabbit Alexa Fluor 647 (Thermo Fisher, A31573), donkey anti-goat Alexa Fluor 488 (Thermo Fisher, A11055), donkey anti-goat Alexa Fluor 633 (Thermo Fisher, A21082), donkey anti-rat Alexa Fluor 488 (Thermo Fisher, A21208), and goat anti-rat Alexa Fluor 647 (Thermo Fisher, A21247). Slides were washed three times with PBS after incubation with secondary antibodies, and mounting medium with 4’,6’-diamidino-2-phenylindole dihydrochloride (DAPI) was added. All images were captured on a Leica SP8 or STELLARIS 5 confocal microscope.

### Bodipy lipid droplet staining

The fixed tissue section that had been incubated with primary and secondary antibodies and fixed cultured fibroblasts were incubated with 2 μM BODIPY (D3922, Invitrogen) staining solution at room temperature for 15 min. After washed three times with PBS, images were captured by using Leica SP8 or STELLARIS 5 confocal microscope.

### Tissue dissociation and FACS

#### Mice

Ten male/ female PDGFRβ-CreER::R26-TdT mice per time points (0 dpi, 5 dpi and 14 dpi) were euthanized by intraperitoneal injection of excess sodium pentobarbital, and then transcardially perfused with phosphate-buffered saline (PBS) solution to remove all the blood. Spinal cord segments (4 mm) centered at the lesion core were isolated and cut into small pieces with sharp scissors. The pieces were digested with papain (LK003176, Worthington Biochemical) containing DNaseI (LK003170, Worthington Biochemical) for 1 h at 37 °C with slow rotation. Then 1.0 mg/mL collagenase type II (LS004176, Worthington Biochemical) and 0.4 mg/mL neutral protease (LS02104, Worthington Biochemical) were added, and the sample was incubated for 30 min at at 37 °C with slow rotation. After centrifugation at 400×*g* for 5 min, the cell pellets were resuspended with 25% Percoll (P1644, Sigma) and centrifuged at 400×*g* for 10 min to remove myelin debris. Using a BD FACSAria Fusion cell sorter, cells were sorted into DPBS buffer according to tdtomato fluorescence. The FSC/SSC was adjusted to isolate single cells, then isolate target cells depending on the expression of tdtomato.

### Rhesus macaques

Monkeys underwent transection SCI (8 mm) and were sacrificed at 7 dpi, 14 dpi, 30 dpi, and 6 mpi. Monkeys were euthanized by intramuscularly injection of ketamine. Single-nucleus RNA-seq was performed on tissues dissected from the lesion edge, while scRNA-seq was performed on tissues from the lesion core. To distinguish between MFs and PFs in normal monkey spinal cord, we removed the meninges of normal monkey prior to scRNA-seq. The method for obtaining single cells was similar to that used for mice. Spinal cord tissues dissociated from monkeys were digested into single cell suspensions using a papain dissociation system (LK003150, Worthington Biochemical)^63^. Debris was removed by Percoll density gradient centrifugation. The method for obtaining single nuclei was based on a published paper^64^. The nuclei were separated from the cells using EZ Lysis Buffer (Sigma), and then cell debris were removed by sucrose density gradient centrifugation. Tissue samples were immersed in ice-cold Nuclei EZ lysis buffer (NUC101-1KT, Sigma) and homogenized by using Dounce tissue grinder (D8938, Sigma). Grind each sample 15 times with pestle A, then 15 times with pestle B. Dilute the lysate with 0.32 M sucrose. After centrifugation at 3200 × *g* for 10 min and discard the supernatant, the precipitate was carefully re-suspended with 0.32 M sucrose. The suspension was then lightly covered with 1 M sucrose and centrifuged for 20 min at 3200×*g*. After the supernatant was discarded, PBS was used to collect the nuclei deposited on the tube wall. After centrifugation at 3200×*g* for 10 min, resuspend cell nuclei with PBS containing 0.01% BSA, and 0.1% RNase inhibitor (2313A, TaKaRa). All operations should be performed on ice or at 2–8 °C.

### Single-cell sequencing and analysis

One sample containing ten spinal cords was collected from PDGFRβ-CreER::R26-TdT mice. Collected cells were passed through the 10× Genomics pipeline (v3) and sequenced on an Illumina HiSeq 4000. Low-quality cells with few genes expressed (<200) and a proportion of mitochondrial genes <0.05 were removed. Several cell types in addition to fibroblasts and pericytes were also observed by integrating data from 0 dpi, 5 dpi, and 14 dpi samples, including astrocytes, oligodendrocytes, endothelial cells, ependymal cells, Schwann cells, myeloid cells, and immune cells. To identify the origin of myofibroblasts, we captured clusters of fibroblasts and pericytes/vSMCs depending on cluster analysis and obtained a total of 33,366 cells. The sequencing files were run through the 10× Cell Ranger 7.0.0 pipeline to generate gene count data, and then Seurat (v3) and our customed R scripts were applied for sequential analyses. The FindAllMarkers function was used for gene differential expression analysis with the Wilcox method and parameters min.pct 0.25 and logfc.threshold 0.25. Using a combination of classical marker expression and calculation of differential gene expression between clusters, we obtained cell type information for all samples.

Ligand–receptor interaction analysis. To explore cell-cell communication and infer potential ligand–receptor interactions between cell types, we applied CellPhoneDB^65^ on annotated Seurat single sample datasets and multi-sample integrated objects. We downloaded a list of all human and mouse orthologous pairs from the Ensembl database. Before loading the gene expression matrix into CellPhoneDB, we replaced mouse gene names with corresponding human orthologous gene names. After obtaining the result files (means and P values) of CellPhoneDB, we generated an R script to extract cell types and ligand–receptor pairs of interest, then cell-cell communication was visualized in a series of dot plots from ggplot2.

DEGs with a *P* value less than 0.05 and log-transformed fold change larger than 0.25 were selected as input. The package clusterProfiler 3.18.1 and database org. Mm.eg.db 3.12.0 were used for GO enrichment analysis. All three ontologies (biological process, cellular component, and molecular function) were exported.

### Isolation and culture of fibroblasts

Two adult SD rats weighting about 250 g were sacrificed by intraperitoneal injection of excess sodium pentobarbital. Spinal cords were dissected, then dura/arachnoid and pia were carefully removed under a stereomicroscope. A cocktail of collagenase type I (C8140, Solarbio) and II (LS004176, Worthington Biochemical) was used for digestion of dura and pia. The dissociated cells were collected in a 15-mL centrifuge tube after filtered through a 100-mm nylon cell strainer. Cell pellets were collected by centrifugation at 500×*g* for 5 min and re-suspended in culture media containing DMEM (C11330, GIBCO), 15% FBS (16000044, GIBCO), and penicillin/streptomycin (15140122, GIBCO).

### Myelin isolation

Two adult SD rats were sacrificed by intraperitoneal injection of excess sodium pentobarbital. Take the telencephalon of two rats, discard the cerebellum and brainstem, place them in a glass dish, and soak with 0.32 M sucrose. After peeling off the surface membrane and blood vessels, cut them into small pieces and place them in a centrifuge tube, and then use a 5 ml pipette to blow and shake. Add 6 mL 0.85 M sucrose into each high-speed centrifuge tube, and then carefully add 2 mL tissue suspension to ensure a clear boundary between the two solutions. After centrifugation at 27,000×rpm for 1 h, the protein was located in the middle separation layer. Add pre cooled water to two 15 ml centrifuge tubes, remove the intermediate layer, place it in pre cooled water, and leave it on ice for 30 min. Resuspending the pellet and store at −80 °C after centrifugation at 12,000×*g* for 30 min. All steps are performed on ice or at 4 °C.

### RT–qPCR

Total RNA of fibroblasts cultured in vitro was extracted using an Ultrapure RNA Kit (CW0581S, CWBIO). Reverse transcription was performed by a Hifair® II 1st Strand cDNA Synthesis Kit (11123ES, YEASEN). RT-qPCR was performed with Hieff® qPCR SYBR® Green Master Mix (11201ES, YEASEN) on a real-time PCR instrument (Bio-RAD). The list of primers used is shown in Table 1.

**Table 1:** List of primers

Emb-F	GCTGGTTGTGCTGAGTTTCA
Emb-R	ATCCCCAGAGTCCGTTTTTC
Crabp2-F	TTCTGGCAACTGGAAGATCA
Crabp2-R	GTTCGAACAGTGGTGGAGGT
Fn1-F	GCGACTCTGACTGGCCTTAC
Fn1-R	CCGTGTAAGGGTCAAAGCAT
Col1a1-F	TGACTGGAAGAGCGGAGAGT
Col1a1-R	GAATCCATCGGTCATGCTCT
Lama1-F	GTGGGCCAGTAAAATCAGGA
Lama1-R	CCGTCAGTGTTTGGATGTTG
Lama2-F	CACAGTGGGGTCCTTGTCTT
Lama2-R	AGTGGACACCTTCCACGTTC
Apod-F	ACCACAACCGAAGGACAAAG
Apod-R	GGACGCAGCTCCTTGTTTAG
Hmgcr-F	CTGGTTCTTGTTCACGCTCA
Hmgcr-R	CCAAAAGCAACGCTAAGCTC
Pmvk-F	TAACATCTGTGCTGTCCTGCG
Pmvk-R	GGACACGCCTTCCACAATCT
Hmgcs1-F	CTGCCTGACTGTGGTTCAGA
Hmgcs1-R	CCCCCATAGCATGCATTAGT
Apoe-F	AACCGCTTCTGGGATTACCT
Apoe-R	TGCGTAGATCCTCCATGTCA
Lipa-F	GGGTGATCTGTTTCGTCGTT
Lipa-R	CACAACTGGTTTGGGACCTT
Soat1-F	TATGCTTTTGGCCAATTTCC
Soat1-R	AAGAATGCGCCATGGATAAG
Abca1-F	CTGGAAGTTTCTGCCCTTTG
Abca1-R	ATGTCGCTCCAGCTCTTTGT

### Endothelial cell tube formation

Matrigel (354230, Corning) was briefly placed in the lower chambers of a 12-well transwell plate (1 mL/well) and incubated for 30 min at 37 °C. Endothelial cells (SCC066, Sigma) were then seeded at a concentration of 5 × 10^5^/mL (800 μL/chamber). PFs or MFs at a density of 1 × 10^5^/mL were cultured in fibroblast culture medium and seeded on the upper chambers (500 μL/chamber). Images were captured to examine the blood vessel formation after 12-h co-culture.

### Quantification and statistical analysis

To characterize TdT+ cells in the uninjured and injured spinal cord of PDGFRβ-CreER::R26-TdT mice, tissue sections from uninjured spinal cord were stained with PDGFRβ, Sox9, Sox10 and Pdx. Tissue sections from injured spinal cord were stained with Desmin, CD31, CD68, Sox9 and Sox10. To calculate the number of TdT+ cells in the lesion core, tissue sections from PDGFRβ-CreER::R26-TdT mice at different time points in the crush and transection SCI models were stained for GFAP, which marked the astrocyte scar border between the lesion core and spared tissue. The number of TdT+ cells in the GFAP-negative area was counted using Adobe Photoshop, while the area of the corresponding section was measured using ImageJ software. To quantify the percentage of dividing cells or Nestin+ cells among TdT+ cells in the lesion core, tissue sections from PDGFRβ-CreER::R26-TdT mice subjected to crush SCI or transection SCI were stained for Ki67 or Nestin at 5 dpi. Cell nuclei were stained by incubating with DAPI.

To characterize TdT+ cells in the uninjured Col1a2-CreER::R26-TdT mice, tissue sections from uninjured spinal cord were stained with PDGFRβ and Col1.

Tissue sections from uninjured Myh11-CreER::R26-TdT mice were stained with CD13, Myh11, Desmin and αSMA. For NG2-CreER::R26-TdT mice, tissue sections from both uninjured and injured spinal cord (14 dpi) were stained with Sox10, Olig2 and Desmin. For Crabp2-CreER::R26-TdT mice, tissue sections from injured spinal cord were stained with Crabp2, Lama1 and Emb. The cell number were counted by Adobe Photoshop.

Emb+ area and Lama1+ area were measured by using ImageJ software. To detect and compare the ability of LA-F and CE-F to phagocytose lipids to form lipid droplets, tissue sections from PDGFRβ-CreER::R26-TdT were stained with Bodipy and Emb. TdT+ cells in Emb+ area are identified as CE-F, while those in Emb- area are LA-F. TdT+ cells containing Bodipy+ signals were defined as TdT+Bodipy+ cells. Adobe Photoshop were used to count the cell number. Col1+ immunofluorescence signal area of PDGFRβ-CreER::R26-TdT mice, Col1a2-CreER::R26-TdT mice, NG2-CreER::R26-TdT mice and Myh11-CreER::R26-TdT were measured by ImageJ software.

All data are reported as mean ± SEM, and *n* values represent the number of mice or samples used in the experiment. Pairwise differences between the two groups were compared using a two-tailed Student’s *t*-test. The remaining data were subjected to one-way or two-way ANOVA as appropriate. Differences were regarded as significant when *P* values were less than 0.05. Statistical analysis was performed using GraphPad Prism 8.

### Reporting summary

Further information on research design is available in the Nature Portfolio Reporting Summary linked to this article.

### Supplementary information

Supplementary Information

Peer Review File

Reporting Summary

### Source data

Source Data

## Data Availability

Source data are provided with this paper. The scRNA-seq raw data of mice generated in this study can be accessed at NCBI GEO accession no. GSE218584. The database is available at: The scRNA-seq raw data of rhesus monkeys can be available in NCBI GEO accession no. GSE228032. The database is available at: https://www.ncbi.nlm.nih.gov/geo/query/acc.cgi?. Source data are provided with this paper.
