# Supplementary material for: Heterogeneous fibroblasts contribute to fibrotic scar formation after spinal cord injury in mice and monkeys
Source: Nat Commun. 2024 Jul 27;15:6321. doi: 10.1038/s41467-024-50564-x (PMC11282111; doi:10.1038/s41467-024-50564-x)
Supplement: Supplementary file 1 — Supplementary Information [file 41467_2024_50564_MOESM1_ESM.pdf]

Supplementary Materials for

Heterogeneous fibroblasts contribute to fibrotic scar formation after spinal cord injury in mice and monkeys

Xiaoyu Xue *et al*.

Corresponding authors: Zhifeng Xiao, zfxiao@genetics.ac.cn; Jianwu Dai, jwdai@genetics.ac.cn; Yannan Zhao, ynzhao@genetics.ac.cn;

Extended data

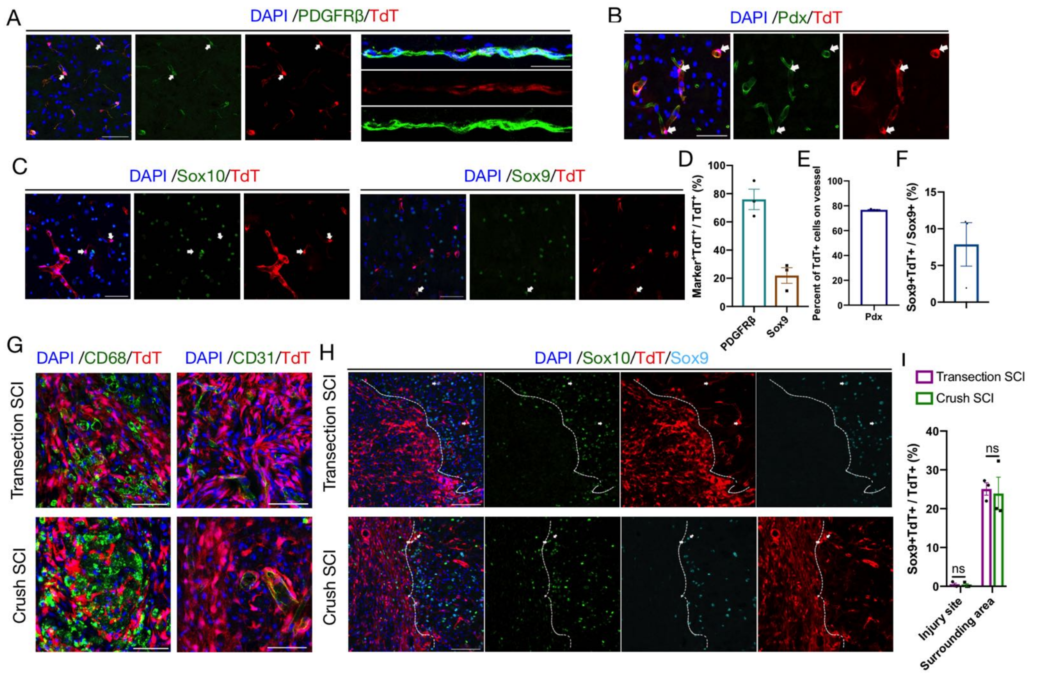

Figure S1 PDGFRβ-CreER::R26-TdT transgenic mice are appropriate to track the cell fate of fibroblasts and pericytes/vSMCs.

(A) Representative images showing that TdT+ cells (red) co-localize with PDGFRβ (green) in the uninjured spinal cord. Arrows indicate the co-localization of TdT and PDGFRβ. Scale bars: 50 μm. Left indicates the parenchyma, and the right indicates the meninges.

(B) PDGFRβ-TdT+ cells (red) are located around vessels (arrows). Endothelial cells are labeled by Pdx (green). Three repeats are performed independently. Arrows indicate the adjacency of TdT and Pdx. Scale bars: 50 μm.

(C) Representative images showing that TdT+ cells (red) co-localize with Sox9 (green, right) but not Sox10 (green, left) in the uninjured spinal cord. Arrows in the left images indicate no co-localization of TdT and Sox10, while arrows in the right images indicate the co-localization of TdT and Sox9. Scale bars: 50 μm.

(D) The percentage of PDGFRβ+TdT+ and Sox9+TdT+ cells in TdT+ cells of the uninjured spinal cord. Data are shown as mean ± SEM. *n* = 3 mice per group.

(E) The percentage of TdT+ cells adhered to vessels in the parenchyma of the uninjured spinal cord. Data are shown as mean ± SEM. *n* = 3 mice per group.

(F) The percentage of Sox9+TdT+ cells in the Sox9+ cells of the uninjured spinal cord. Data are shown as mean ± SEM. *n* = 3 mice per group.

(G) Spinal cord sections from PDGFRβ-CreER::R26-TdT mice were stained with microglia/macrophages markers CD68 (green) and endothelial cells marker CD31 (green). After transection SCI (top) or crush SCI (bottom), PDGFRβ-TdT+ cells (red) in the lesion core were not positive for CD68 or CD31. Three repeats are performed independently. Scale bars: 50 μm.

(H) Representative images showing the expression of Sox9 (light blue) and Sox10 (green) at 14 dpi in PDGFRβ-CreER::R26-TdT mice. Arrows indicating the co-localization of TdT+ (red) with Sox9 in the surrounding area of injury site after transection SCI or crush SCI. Scale bars: 100 μm.

(I) The percentage of Sox9+TdT+ cells in TdT+ cells in the surrounding area and injury site after transection SCI or crush SCI. Data are shown as mean ± SEM. *n* = 3 mice per group. ns, non-significant by two-sided, unpaired Student's *t*-test. *P* = 0.861418 (injury site), *P* = 0.808447 (surrounding area). Source data are provided as a Source Data file.

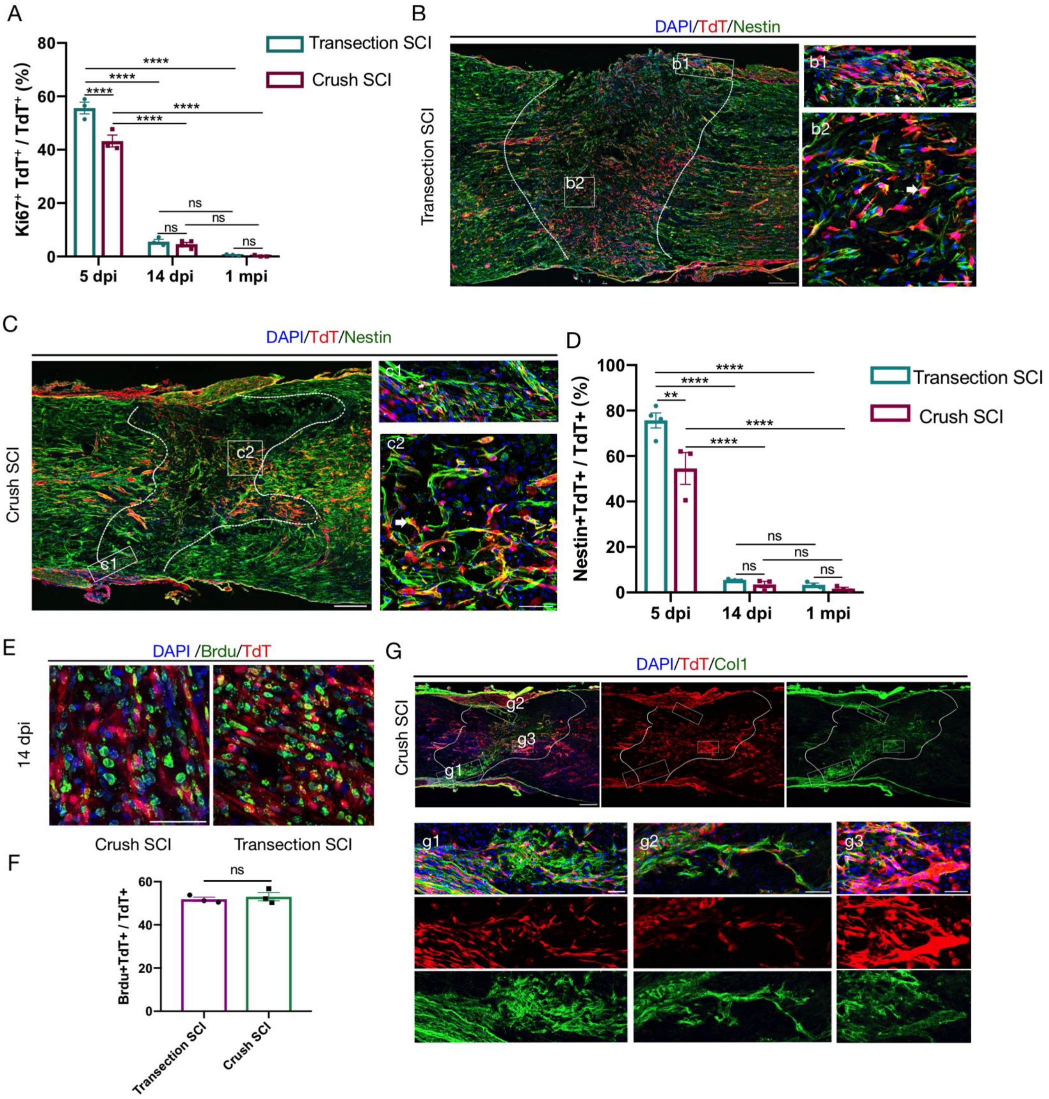

Figure S2 PDGFRβ+ cells proliferate and express Nestin after SCI and contribute to the formation of fibrotic scars.

(A) Quantification of the percentage of proliferating PDGFRβ-TdT+ cells, as determined by co-expression of Ki67 and TdT. Data are shown as mean ± SEM. *n* = 3 mice per group. ns = 0.9340 (transection SCI vs. crush SCI at 14 dpi). ns = 0.9965 (transection SCI vs. crush SCI at 1 mpi). ns = 0.0591 (14 dpi vs. 1mpi in transection SCI), ns = 0.0783 (14 dpi vs. 1mpi in crush SCI). ****$P < 0.0001$ by two-way ANOVA with Sidak's multiple comparisons test.

(B-C) Representative images showing that in the transection (A) and crush (B) SCI models, PDGFRβ-TdT+ cells (red) contribute to the Nestin+ cells (green) in the lesion core at 5 dpi. b1–b2 and c1–c2, magnified boxed regions in B and

C, respectively. Arrows showing the co-localization of TdT and Nestin. Dashed lines label the lesion sites. Scale bars: 250 μm (B and C), 50 μm (b1–b2, c1–c2).

(D) Quantification of the percentage of Nestin+TdT+ cells in TdT+ cells of the lesion core. Dashed lines label the lesion sites. $n = 4$ mice at 5 dpi of transection SCI. $n = 3$ mice per group in other groups. Data are shown as mean ± SEM. **$P = 0.0011$, ****$P < 0.0001$ by two-way ANOVA with Sidak's multiple comparisons test.

(E) Representative images showing the co-labeling of TdT (red) and BrdU (green) after transection SCI or crush SCI. Scale bars: 50 μm.

(F) The percentage of BrdU+TdT+ cell in TdT+ cells in the transection SCI or crush SCI model. Data are shown as mean ± SEM. $n = 3$ mice per group. ns, non-significant by two-sided, unpaired Student's t-test. $P = 0.6077$.

(G) Representative confocal images showing the continuity of Col1+ signal (green) from the meninges to parenchyma at 5 dpi. g1–g3 represent magnified images of boxed areas in G. Scale bars: 250 μm, 50 μm (g1–g3). Dashed lines label the lesion sites. Three repeats are performed independently. Source data are provided as a Source Data file.

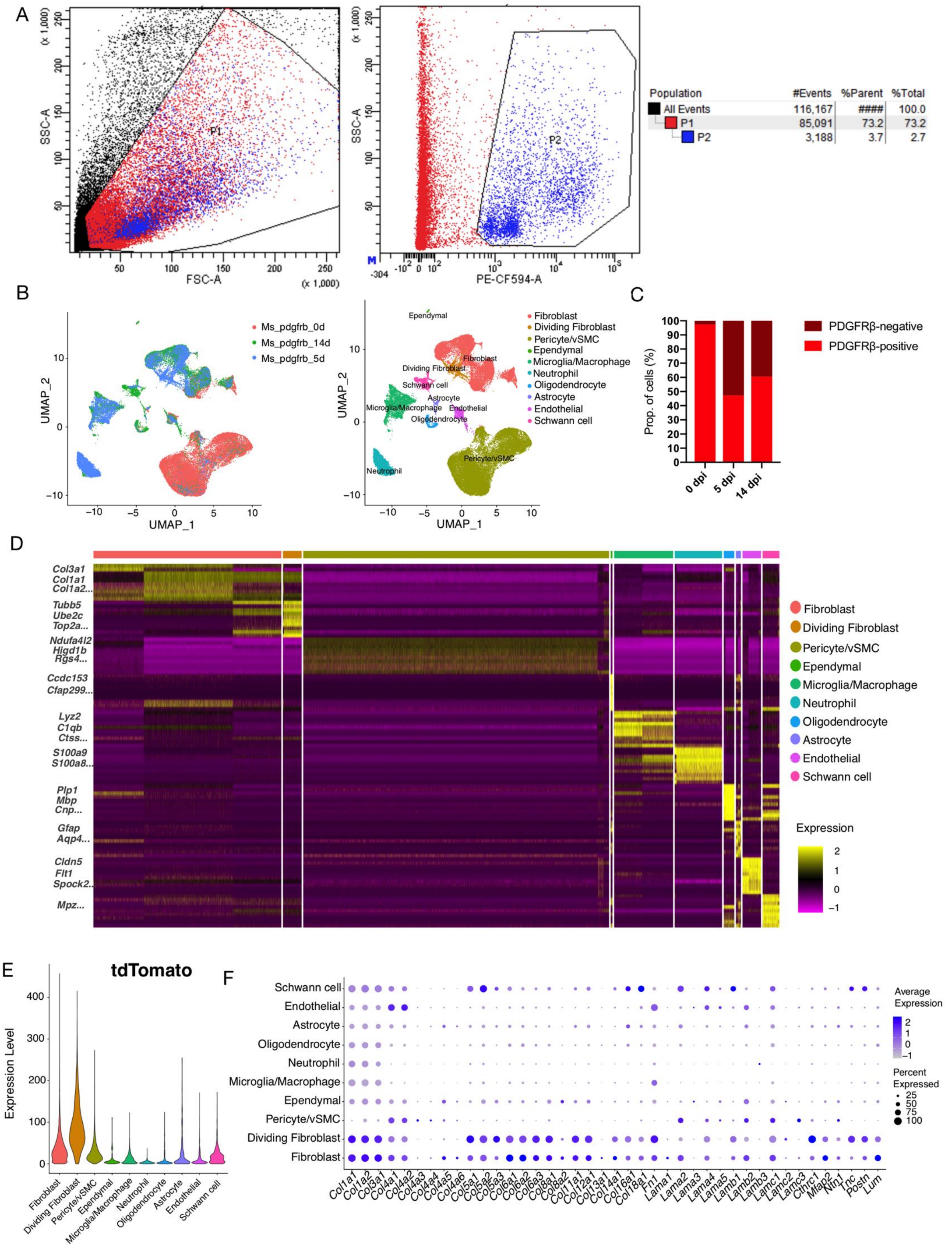

Figure S3 Major cell types obtained from uninjured and transected mouse spinal cords.

(A) PDGFRβ-TdT+ cells from uninjured and injured spinal cords were purified by FACS for single-cell RNA sequencing.

(B) UMAP plots showing the identities of the major cell types obtained from uninjured and injured spinal cords at different time points.

(C) Bar graph showing the proportion of PDGFRβ-positive cells and PDGFRβ-negative cells relative to the total number of cells at 0 dpi, 5 dpi, and 14 dpi.
(D) Heatmap showing the expression patterns of the top 10 differentially expressed genes in all clusters.
(E) Violin plot showing the expression of tdtomato (TdT) in different cells identified in (B).
(F) Dot plot of ECM-associated genes showing the higher expression level of ECM-associated genes in fibroblasts and pericytes/vSMCs. Source data are provided as a Source Data file.

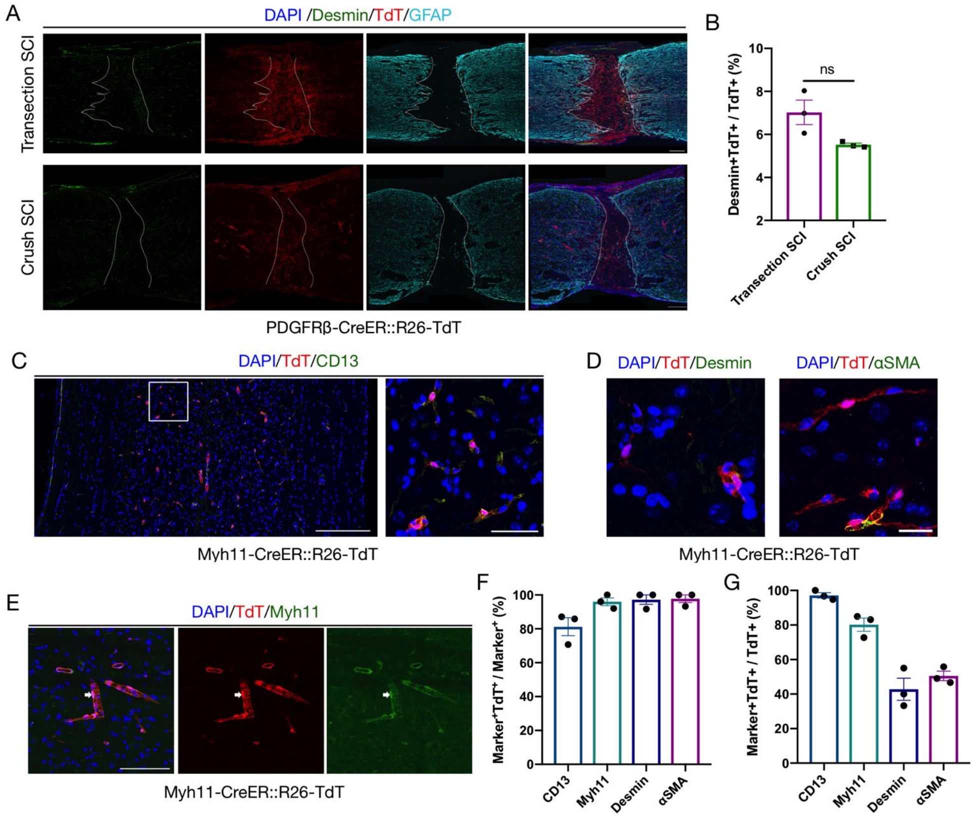

Figure S4 Pericytes/vSMCs are labeled in Myh11-CreER:R26-TdT mice.

(A) Representative images showing the expression of TdT (red) and Desmin (green) in PDGFRβ-CreER::R26-TdT mice after transection or crush SCI. Dashed lines label the lesion sites. Scale bar: 250 μm.

(B) The percentage of Desmin+TdT+ cells in TdT+ cells in the injury site of transection SCI and crush SCI model. Data are shown as mean ± SEM. n = 3 mice per group. ns, non-significant by two-sided, unpaired Student's *t*-test. P = 0.0593.

(C) Representative image showing the co-localization of TdT (red) and CD13 (green) in Myh11-CreER::R26-TdT mice. Right panel showing the magnification image of boxed region of C. Scale bar: 250 μm, 50 μm (enlarged view).

(D) Representative image showing the co-localization of TdT (red) and Desmin (green), as well as TdT (red) and αSMA (green) in Myh11-CreER::R26-TdT mice. Scale bar: 20 μm.

(E) Representative image showing the co-localization of TdT (red) and Myh11 (green) in Myh11-CreER::R26-TdT mice. Arrows indicate the co-localization of TdT and Myh11. Scale bar: 100 μm.

(F) The percentage of TdT+ cells in CD13+, Myh11+, Desmin+ and αSMA+ cells. *n* = 3 mice per group. Data are expressed as mean ± SEM.

(G) Percentage of CD13+, Myh11+, Desmin+ and αSMA+ cells in TdT+ cells. *n* = 3 mice per group. Data are expressed as mean ± SEM. Source data are provided as a Source Data file.

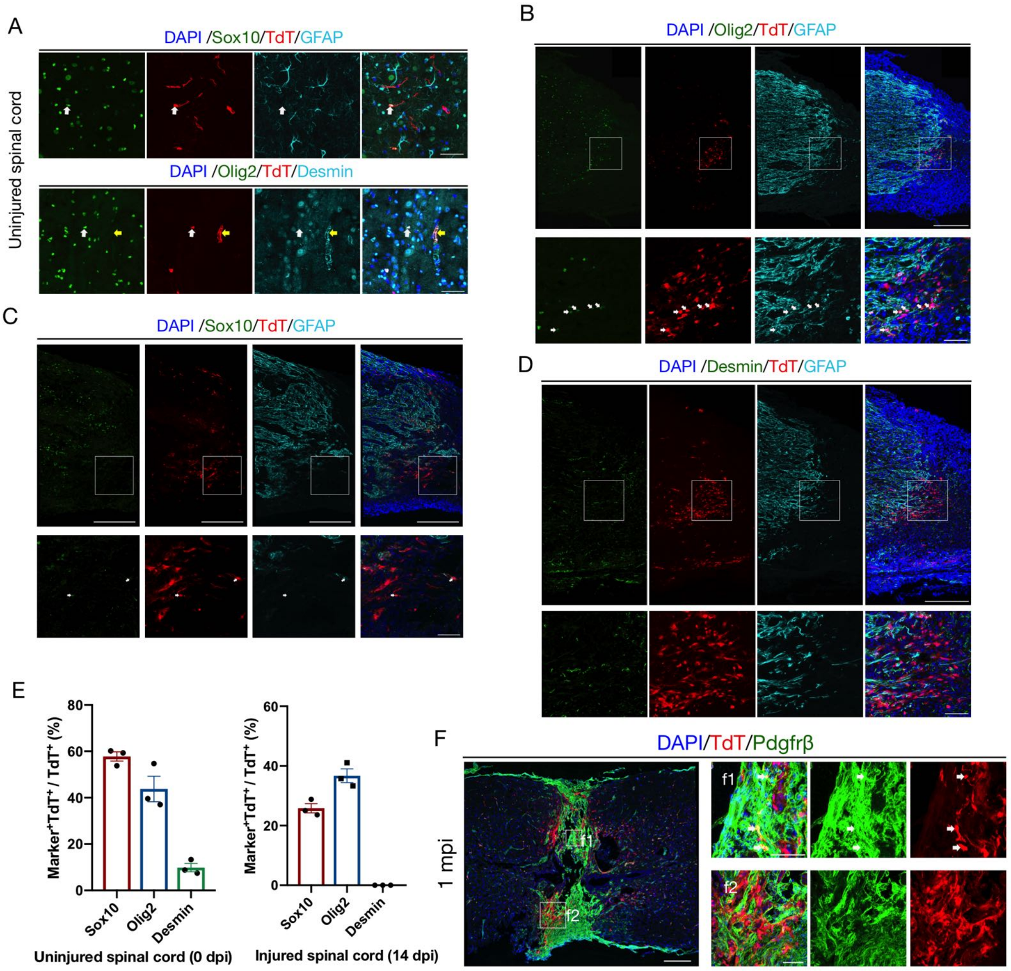

Figure S5 NG2+ Pericytes/vSMCs are not the major origin of fibrotic scar.

(A) Representative confocal images showing the co-localization of TdT (red) with Sox10 (green), Olig2 (green) and Desmin (light blue) in the uninjured spinal cord of NG2-CreER::R26-TdT mice. In upper images, white arrows indicate the co-localization of TdT and Sox10 rather than with GFAP, while in lower images, white arrows indicate co-localization of TdT with Olig2 but not Desmin, and yellow arrows in lower images indicate co-localization of TdT with Desmin but not Olig2. Scale bar: 50 μm.

(B-D) Representative confocal images showing the co-localization of TdT (red) with Olig2 (green in B), Sox10 (green in C) and Desmin (Green in D) in NG2-CreER::R26-TdT mice after transection SCI at 14 dpi. The rectangular boxes indicate the enlarged regions in the images below. Arrows indicate the co-localization of TdT and Olig2 in figure B. Arrows indicate the co-localization of TdT and Sox10 in figure C. Scale bar: 250 μm, 50 μm (enlarged view).

(E) The percentage of Sox10+TdT+, Olig2+TdT+ and Desmin+TdT+ cells in NG2-TdT+ cells in the uninjured spinal cord and injured spinal cord. Data are shown as mean ± SEM. *n* = 3 mice per group.

(F) Representative images of spinal cord sections of NG2-CreER::R26-TdT mice at 1 mpi stained with an antibody against PDGFRβ. f1, f2, higher-magnification images of boxed regions in F. Arrows indicate the co-localization of TdT (red) and PDGFRβ (green). Three repeats are performed independently. Scale bar: 250 μm, 50 μm (f1, f2). Source data are provided as a Source Data file.

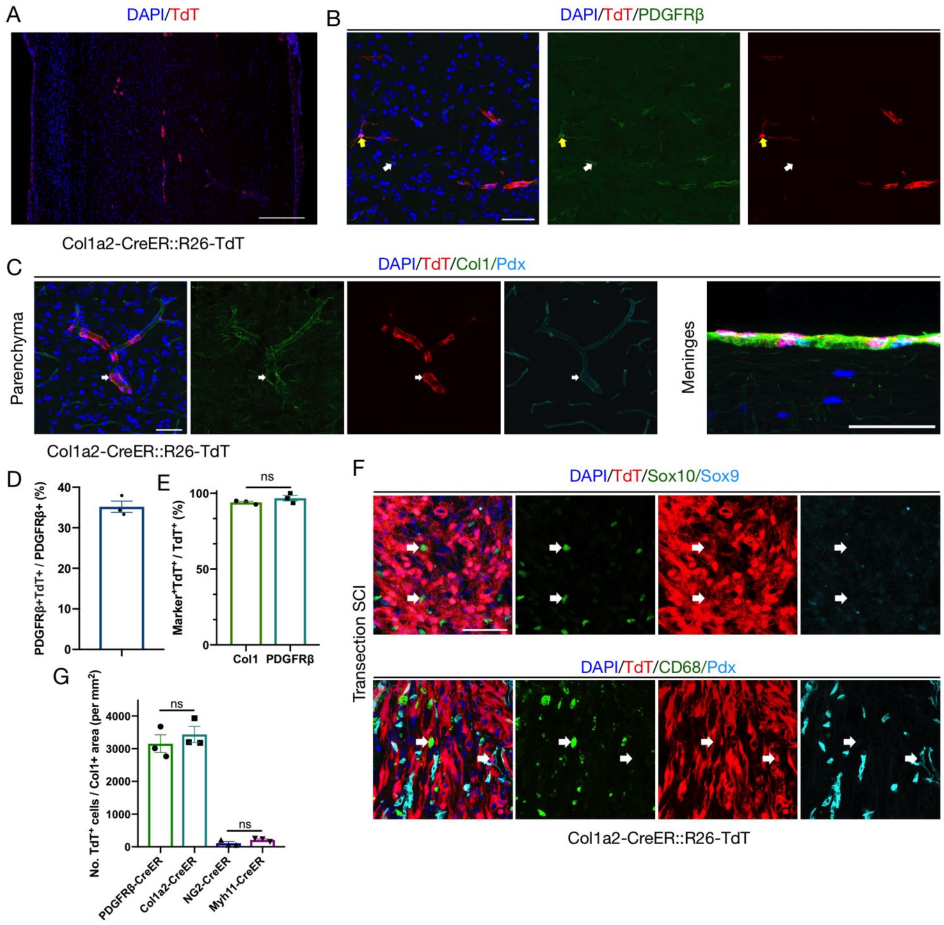

Figure S6 Col1+ fibroblasts were specifically labeled in Col1a2-CreER::R26-TdT mice.

(A) Representative image showing the expression of TdT+ cells (red) in uninjured spinal cord parenchyma and meninges of Col1a2-CreER::R26-TdT mice. Scale bar: 250 μm.

(B) Representative images showing the expression of TdT (red) and PDGFRβ (green) in uninjured spinal cord parenchyma of Col1a2-CreER::R26-TdT mice. Yellow arrows indicating the co-localization of TdT and PDGFRβ, White arrows indicating the PDGFRβ+ TdT- cell. Scale bar: 50 μm.

(C) Representative images showing the co-localization of TdT (red) and Col1 (green) in uninjured spinal cord parenchyma and meninges of Col1a2-CreER::R26-TdT mice. In parenchyma, while arrows indicate TdT+Col1a2+ cells located attached to Pdx+ (light blue) blood vessels. Scale bar: 50 μm.

(D) The percentage of PDGFRβ+TdT+ cells in PDGFRβ+ cells in the uninjured spinal cord parenchyma of Col1a2-CreER::R26-TdT mice. Data are shown as mean ± SEM. *n* = 3 mice per group.

(E) The percentage of Col1+ cells and PDGFRβ+ cells in TdT+ cells in the uninjured spinal cord parenchyma of Col1a2-CreER::R26-TdT mice. Data are shown as mean ± SEM. *n* = 3 mice per group. ns, non-significant by two-sided, unpaired Student's *t*-test. *P* = 0.2263.

(F) In the transection SCI model of Col1a2-CreER::R26-TdT mice, TdT+ (red) was not expressed in oligodendrocytes (Sox10, green), astrocytes (Sox9, light blue), macroglia/macrophages (CD68, green) and endothelial cells (Pdx, light blue). Arrows indicate no co-localization of TdT with Sox10, Sox9 or CD68. Scale bar: 50 μm.

(G) The densities of TdT+ cells in the Col1+ area in different transgenic mice, including PDGFRβ-CreER::R26-TdT mice, Col1a2-CreER::R26-TdT, NG2-CreER::R26-TdT mice and Myh11-CreER::R26-TdT mice. Data are shown as mean ± SEM. *n* = 3 mice per group. ns = 0.7111 (PDGFRβ-CreER::R26-TdT vs. Col1a2-CreER::R26-TdT), ns = 0.9804 (NG2-CreER::R26-TdT vs. Myh11-CreER::R26-TdT) by one-way ANOVA with Tukey's multiple comparisons test. Source data are provided as a Source Data file.

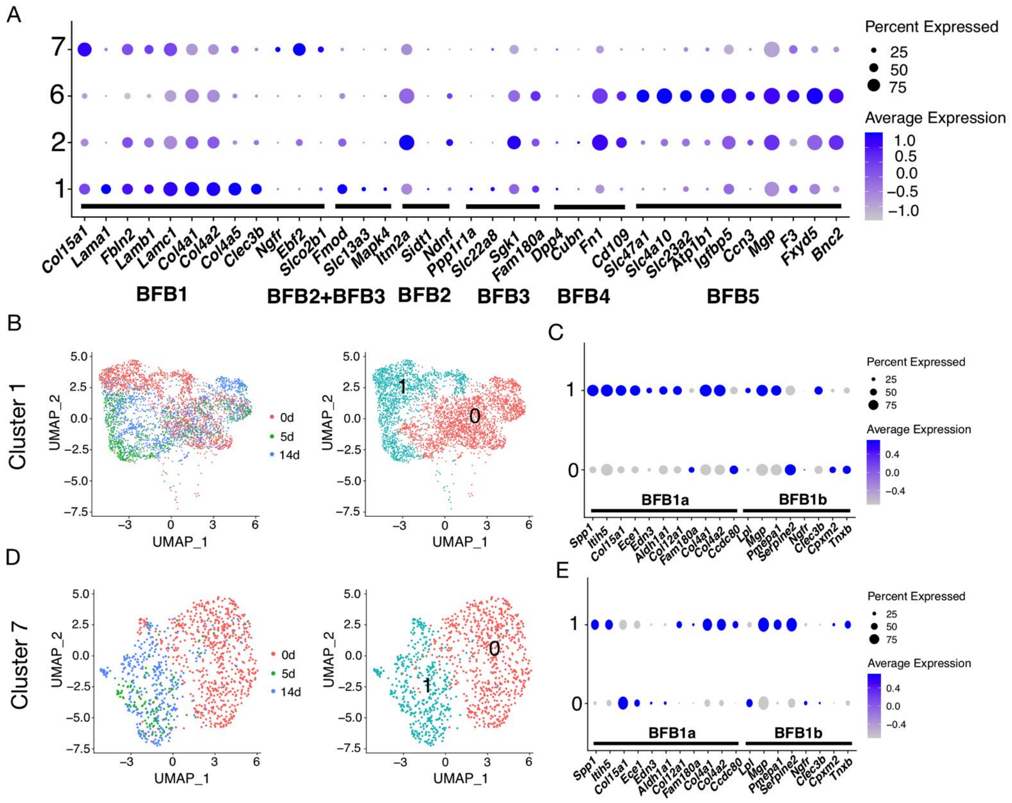

Figure S7 Spinal cord fibroblasts annotation depending on the marker genes of BFB1-5[1].

(A) Dot plot showing the expression pattern of BFB1-5 marker genes in spinal cord fibroblasts.

(B) UMAP plots of re-clustered cells of cluster 1 spinal cord fibroblasts in figure 3B.

(C) Dot plot showing the expression of selected marker genes used to identify BFB1a and BFB1b in the two subtypes of cluster 1.

(D) UMAP plots of re-clustered cells of cluster 7 spinal cord fibroblasts in figure 3B.

(E) Dot plot showing the expression of selected marker genes used to identify BFB1a and BFB1b in the two subtypes of cluster 7.

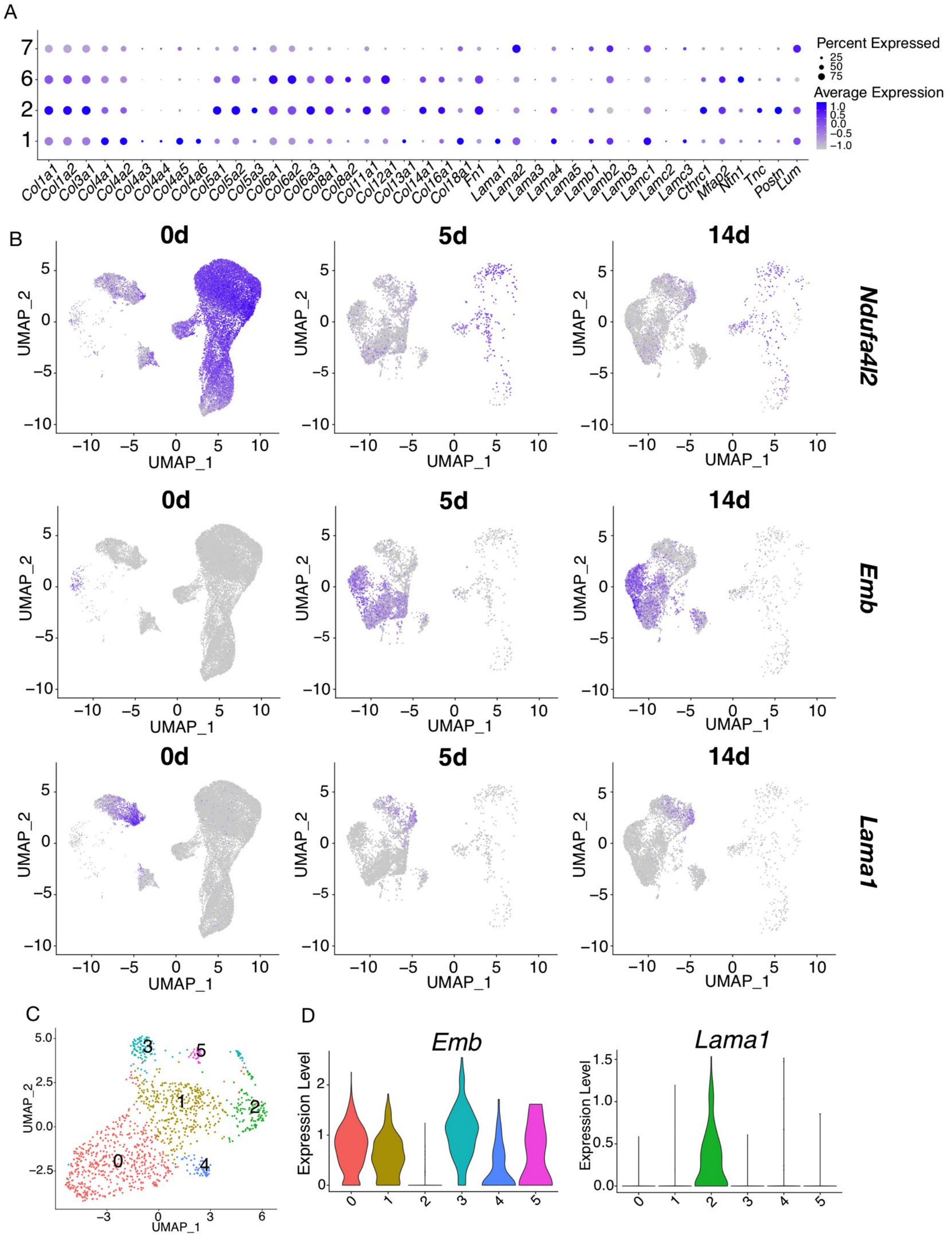

Figure S8 Extended figures for gene expression in fibroblasts of mice.

(A) Dot plot showing the expression of ECM related genes in fibroblasts identified in figure 3B, suggesting similarities between clusters 2 and 6.

(B) Expression pattern of marker genes of pericytes/vSMCs, PFs and MFs.

(C) Dividing fibroblasts were extracted and reclustered in new UMAP coordinates.

(D) Violin plot showing the expression patterns of Emb and Lama1 in dividing fibroblasts.

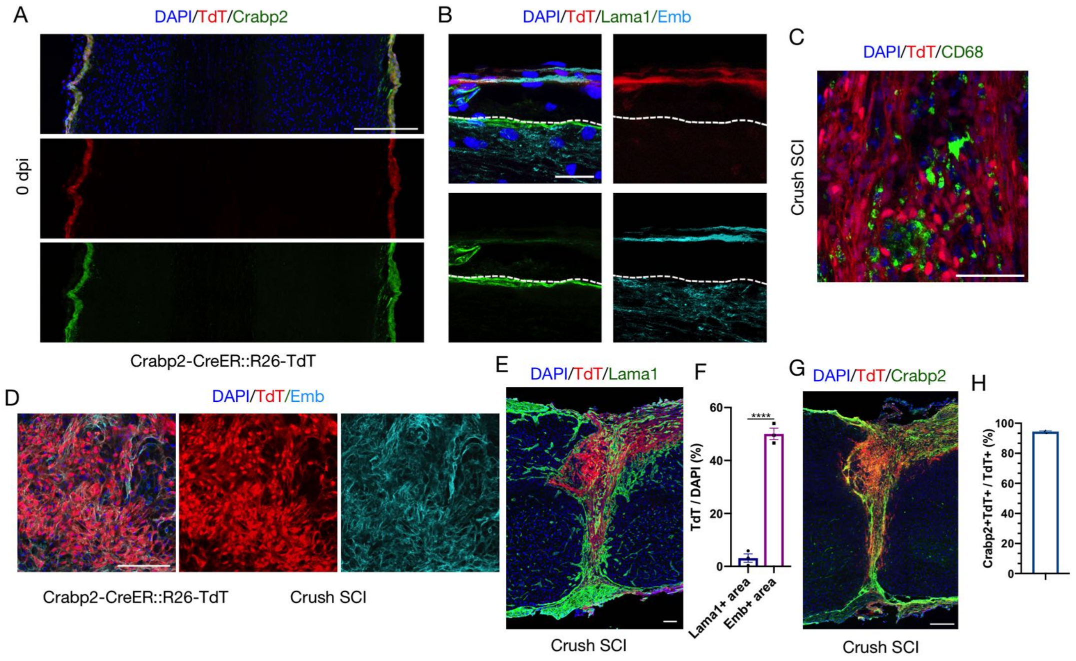

Figure S9 Crabp2-TdT+ cells express Emb and Crabp2 in both uninjured and injured spinal cord.

(A) Representative images showing the expression of TdT (red) and Crabp2 (green) in the meninges of Crabp2-CreER::R26-TdT mice. Three repeats are performed independently. Scale bar: 250 μm.

(B) In uninjured Crabp2-CreER::R26-TdT mice, TdT+ cells (red) were distributed in Emb+ dura (light blue), but not Lama1+ pia (green). Three repeats are performed independently. Dashed lines distinguish dura/arachnoid and pia mater. Scale bar: 25 μm.

(C) After SCI, Crabp2-TdT (red) was not expressed in CD68+ microglia/macrophages (green). Three repeats are performed independently. Scale bar: 50 μm.

(D) After SCI, Crabp2-TdT+ cells (red) expressed Emb (light blue) in the lesion core. Three repeats are performed independently. Scale bar: 50 μm.

(E) After SCI, Crabp2-TdT+ cells (red) were distributed inner of Lama1+ area (green). Scale bar: 100 μm.

(F) Quantitative statistics showing the ratio of Crabp2-TdT+ cells in Emb+ area and Lama1+ area. Data are shown as mean ± SEM. n = 3 mice per group. ****p < 0.0001 by by two-sided, unpaired Student's *t*-test.

(G) Representative image showing the co-localization of Crabp2 (green) and TdT (red) at 14 dpi in Crabp2-CreER::R26-TdT mice.

(H) Quantitative statistics showing the percentage of Crabp2+TdT+ cells in TdT+ cells. Data are shown as mean ± SEM. *n* = 3 mice per group. Source data are provided as a Source Data file.

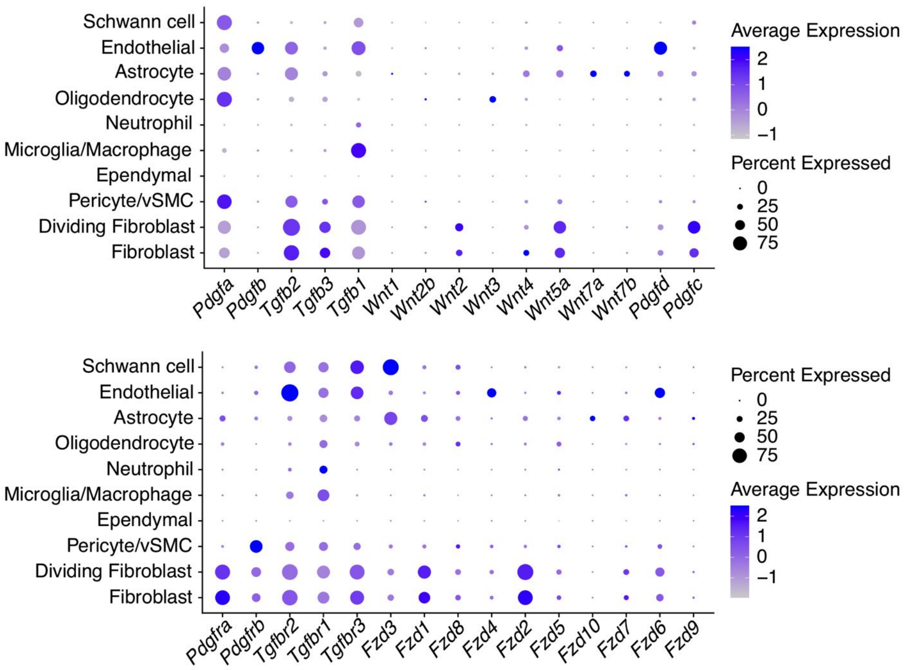

Figure S10 The expression of ligands and receptors of TGF-β, Wnt and PDGF pathways in different cells at 5 dpi in mouse transection SCI model. Dot plot showing the expression of ligand genes and receptor genes related to TGF-β, Wnt and PDGF pathways in many cell types, including Schwann cells, endothelial cells, astrocytes, oligodendrocytes, neutrophils, macroglia/macrophages, ependymal cells, pericytes/vSMCs, dividing fibroblasts and fibroblasts.

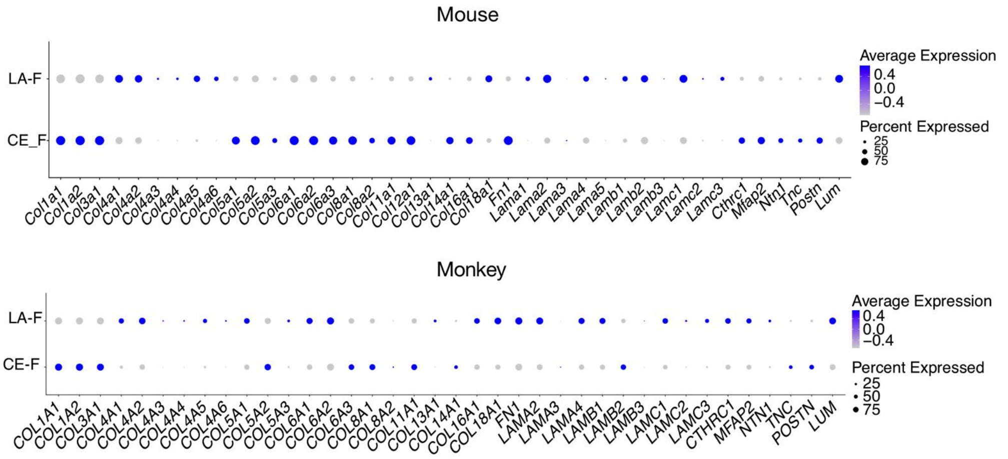

Figure S11 The expression patterns of ECM-related genes in LA-F and CE-F are similar between mice and monkeys.

Dot plot showing that mouse and monkey CE-F express higher levels of collagen I and Fn1, while LA-F express higher levels of collagen IV and laminin.

Reference

1. Pietilä, R., *et al.* Molecular anatomy of adult mouse leptomeninges. *Neuron* **111**, 3745-3764.e3747 (2023).
