# Supplementary material for: Heterogeneous fibroblasts contribute to fibrotic scar formation after spinal cord injury in mice and monkeys
Source: Nat Commun. 2024 Jul 27;15:6321. doi: 10.1038/s41467-024-50564-x (PMC11282111; doi:10.1038/s41467-024-50564-x)
Supplement: Supplementary file 3 — Reporting Summary [file 41467_2024_50564_MOESM3_ESM.pdf]

nature portfolio

Corresponding author(s): Yannan Zhao, Jianwu Dai, Zhifeng Xiao

Last updated by author(s): Jun 10, 2024

Reporting Summary

Nature Portfolio wishes to improve the reproducibility of the work that we publish. This form provides structure for consistency and transparency in reporting. For further information on Nature Portfolio policies, see our Editorial Policies and the Editorial Policy Checklist.

Statistics

For all statistical analyses, confirm that the following items are present in the figure legend, table legend, main text, or Methods section.

n/a	Confirmed	
☐	☒	The exact sample size (*n*) for each experimental group/condition, given as a discrete number and unit of measurement
☐	☒	A statement on whether measurements were taken from distinct samples or whether the same sample was measured repeatedly
☐	☒	The statistical test(s) used AND whether they are one- or two-sided<br>*Only common tests should be described solely by name; describe more complex techniques in the Methods section.*
☒	☐	A description of all covariates tested
☐	☒	A description of any assumptions or corrections, such as tests of normality and adjustment for multiple comparisons
☐	☒	A full description of the statistical parameters including central tendency (e.g. means) or other basic estimates (e.g. regression coefficient) AND variation (e.g. standard deviation) or associated estimates of uncertainty (e.g. confidence intervals)
☐	☒	For null hypothesis testing, the test statistic (e.g. *F*, *t*, *r*) with confidence intervals, effect sizes, degrees of freedom and *P* value noted<br>*Give P values as exact values whenever suitable.*
☒	☐	For Bayesian analysis, information on the choice of priors and Markov chain Monte Carlo settings
☒	☐	For hierarchical and complex designs, identification of the appropriate level for tests and full reporting of outcomes
☒	☐	Estimates of effect sizes (e.g. Cohen's *d*, Pearson's *r*), indicating how they were calculated

Our web collection on statistics for biologists contains articles on many of the points above.

Software and code

Policy information about availability of computer code

Data collection	Leica SP8 and Leica STELLARIS 5 microscope/software were used for confocal image acquisition. Flow cytometry was performed by using BD FACSAria Fusion cell sorter. Single-cell RNA-seq was performed on 10x Genomics platform.
Data analysis	Adobe photoshop CC and Image J was used for cell counts and area measurement. Statistical analysis was performed by Microsoft Excel 16.54 and Prism 8 for MAC. Single-cell RNA-seq analysis was performed bySeurat (v3) and customed R scripts. CellPhoneDB was used for Ligand–receptor interaction analysis.

For manuscripts utilizing custom algorithms or software that are central to the research but not yet described in published literature, software must be made available to editors and reviewers. We strongly encourage code deposition in a community repository (e.g. GitHub). See the Nature Portfolio guidelines for submitting code & software for further information.

Data

Policy information about availability of data

All manuscripts must include a data availability statement. This statement should provide the following information, where applicable:

- Accession codes, unique identifiers, or web links for publicly available datasets
- A description of any restrictions on data availability
- For clinical datasets or third party data, please ensure that the statement adheres to our policy

The scRNA-seq raw data of mice generated in this study can be accessed at NCBI GEO accession no. GSE218584. The database is available at:https://www.ncbi.nlm.nih.gov/geo/query/acc.cgi?acc=GSE218584. The sequencing datasets of rhesus monkeys can be available in figshare: https://doi.org/10.6084/

m9.figshare.22068830.v1.

Human research participants

Policy information about studies involving human research participants and Sex and Gender in Research.

Reporting on sex and gender	*Use the terms sex (biological attribute) and gender (shaped by social and cultural circumstances) carefully in order to avoid confusing both terms. Indicate if findings apply to only one sex or gender; describe whether sex and gender were considered in study design whether sex and/or gender was determined based on self-reporting or assigned and methods used. Provide in the source data disaggregated sex and gender data where this information has been collected, and consent has been obtained for sharing of individual-level data; provide overall numbers in this Reporting Summary. Please state if this information has not been collected. Report sex- and gender-based analyses where performed, justify reasons for lack of sex- and gender-based analysis.*
Population characteristics	*Describe the covariate-relevant population characteristics of the human research participants (e.g. age, genotypic information, past and current diagnosis and treatment categories). If you filled out the behavioural & social sciences study design questions and have nothing to add here, write "See above."*
Recruitment	*Describe how participants were recruited. Outline any potential self-selection bias or other biases that may be present and how these are likely to impact results.*
Ethics oversight	*Identify the organization(s) that approved the study protocol.*

Note that full information on the approval of the study protocol must also be provided in the manuscript.

Field-specific reporting

Please select the one below that is the best fit for your research. If you are not sure, read the appropriate sections before making your selection.

[x] Life sciences [ ] Behavioural & social sciences [ ] Ecological, evolutionary & environmental sciences

For a reference copy of the document with all sections, see nature.com/documents/nr-reporting-summary-flat.pdf

Life sciences study design

All studies must disclose on these points even when the disclosure is negative.

Sample size	Sample size was determined by our previous experience. 3 to 4 animals per group were sufficient to obtain statistically significant results.
Data exclusions	No data were excluded from the analysis.
Replication	Quantitative statistics of cell characteriazation on tissure sections were perfomed by using 2-4 tissue sections from at least 3 mice. All attempts on replication were successful.
Randomization	Animals which were chosen to carry out experiments were random.
Blinding	Investigators were blinded to group allocation during data collection and/or analysis.

Reporting for specific materials, systems and methods

We require information from authors about some types of materials, experimental systems and methods used in many studies. Here, indicate whether each material, system or method listed is relevant to your study. If you are not sure if a list item applies to your research, read the appropriate section before selecting a response.

Materials & experimental systems

n/a	Involved in the study	
[ ]	[x]	Antibodies
[ ]	[x]	Eukaryotic cell lines
[x]	[ ]	Palaeontology and archaeology
[ ]	[x]	Animals and other organisms
[x]	[ ]	Clinical data
[x]	[ ]	Dual use research of concern

Methods

n/a	Involved in the study	
[x]	[ ]	ChIP-seq
[ ]	[x]	Flow cytometry
[x]	[ ]	MRI-based neuroimaging

Antibodies

Antibodies used

CD31 (1:200, rat, BD Biosciences, 553370, clone MEC 13.3), Podocalyxin (1:500, goat, R&D Systems, AF1556-SP), Vimentin (1:500, rabbit, Abcam, ab92547, clone EPR3776), PDGFRβ (1:250, rabbit, Abcam, ab32570, clone Y92; 1:100, rat, Invitrogen, 14-1402-82, clone APB5), GFAP (1:500, rabbit, Abcam, ab7260; 1:500, chicken, Abcam, ab4674; 1:500, rat, Invitrogen, 13-0300, clone 2.2B10), Ki67 (1:500, rabbit, Abcam, ab15580), Nestin (1:200, chicken, Abcam, ab134017), Col1 (1:250, rabbit, Abcam, ab21286), Fibronectin (1:250, rabbit, Sigma, F3648; 1:250, rabbit, Abcam, ab23750), NG2 (1:200, rabbit, Sigma, AB5320), CD68 (1:500, rabbit, Abcam, ab125212), Lama1 (1:500, rabbit, Sigma, L9393/AB2034), Emb (1:100, rat, Invitrogen, 14-5839-81, clone G7.43.1),Crabp2 (1:200, rabbit, Proteintech, 10225-1-AP), Lama2 (1:200, rat, Sigma, L0663,), Acta2 (1:200, rabbit, Abcam, ab5694), Col6a1 (1:200, rabbit, Proteintech, 17023-1-AP), Sox9 (1:500, goat, R&D Systems, AF3075), Sox10 (1:500, rabbit, Abcam, ab155279), BrdU (1:500, rat, Abcam, ab6326), Desmin (1:500, rabbit, Abcam, ab15200), Olig2 (1:500, rabbit, Abcam, ab109186), CD13 (1:500, rat, Abcam, ab33489) and Myh11 (1:500, mouse, Santa Cruz, sc-6956). goat anti-chicken Alexa Fluor 488 (Thermo Fisher, A11039), donkey anti-rabbit Alexa Fluor 488 (Thermo Fisher, A21206), donkey anti-rabbit Alexa Fluor 647 (Thermo Fisher, A31573), donkey anti-goat Alexa Fluor 488 (Thermo Fisher, A11055), donkey anti-goat Alexa Fluor 633 (Thermo Fisher, A21082), donkey anti-rat Alexa Fluor 488 (Thermo Fisher, A21208), goat anti-rat Alexa Fluor 647 (Thermo Fisher, A21247).

Validation

The antibodies have been validated in published papers.
CD31 (1:200, rat, BD Biosciences, 553370, clone MEC 13.3), Invitrogen, 14-1402-82, clone APB5), GFAP (1:500, rabbit, Abcam, ab7260), Col1 (1:250, rabbit, Abcam, ab21286): Dorrier, C.E., Aran, D., Haenelt, E.A., Sheehy, R.N., Hoi, K.K., Pintarić, L., Chen, Y., Lizama, C.O., Cautivo, K.M., Weiner, G.A., et al. (2021). CNS fibroblasts form a fibrotic scar in response to immune cell infiltration. Nature neuroscience 24, 234-244.

Podocalyxin (1:500, goat, R&D Systems, AF1556-SP), Vimentin (1:500, rabbit, Abcam, ab92547, clone EPR3776), PDGFRβ (1:250, rabbit, Abcam, ab32570, clone Y92; 1:100, rat), NG2 (1:200, rabbit, Sigma, AB5320): Dias, D.O., Kalkitsas, J., Kelahmetoglu, Y., Estrada, C.P., Tatarishvili, J., Holl, D., Jansson, L., Banitalebi, S., Amiry-Moghaddam, M., Ernst, A., et al. (2021). Pericyte-derived fibrotic scarring is conserved across diverse central nervous system lesions. Nature communications 12, 5501.

Fibronectin (1:250, rabbit, Sigma, F3648): Tsata, V., Möllmert, S., Schweitzer, C., Kolb, J., Möckel, C., Böhm, B., Rosso, G., Lange, C., Lesche, M., Hammer, J., et al. (2021). A switch in pdgfrb(+) cell-derived ECM composition prevents inhibitory scarring and promotes axon regeneration in the zebrafish spinal cord. Developmental cell 56, 509-524.e509.

GFAP (1:500, rat, Invitrogen, 13-0300, clone 2.2B10), Lama1 (1:500, rabbit, Sigma, L9393): Li, Y., He, X., Kawaguchi, R., Zhang, Y., Wang, Q., Monavarfeshani, A., Yang, Z., Chen, B., Shi, Z., Meng, H., et al. (2020). Microglia-organized scar-free spinal cord repair in neonatal mice. Nature 587, 613-618.

GFAP (1:500, chicken, Abcam, ab4674): Milich, L.M., Choi, J.S., Ryan, C., Cerqueira, S.R., Benavides, S., Yahn, S.L., Tsoulfas, P., and Lee, J.K. (2021). Single-cell analysis of the cellular heterogeneity and interactions in the injured mouse spinal cord. The Journal of experimental medicine 218, doi:10.1084/jem.20210040.

Higher citation in website CiteAb (https://www.citeab.com):
Ki67 (1:500, rabbit, Abcam, ab15580), Nestin (1:200, chicken, Abcam, ab134017), Fibronectin (1:250, rabbit, Abcam, ab23750), CD68 (1:500, rabbit, Abcam, ab125212) , Emb (1:100, rat, Invitrogen, 14-5839-81, clone G7.43.1), Lama2 (1:200, rat, Sigma, L0663,), Acta2 (1:200, rabbit, Abcam, ab5694), Col6a1 (1:200, rabbit, Proteintech, 17023-1-AP), Sox9 (1:500, goat, R&D Systems, AF3075), Sox10 (1:500, rabbit, Abcam, ab155279), BrdU (1:500, rat, Abcam, ab6326), Desmin (1:500, rabbit, Abcam, ab15200), Myh11 (1:500, mouse, Santa Cruz, sc-6956).

Eukaryotic cell lines

Policy information about cell lines and Sex and Gender in Research

Cell line source(s)

The endothelial cell line was derived from human temporal lobe microvessels removed during epilepsy control surgery.

Authentication

DNA was extracted by genomic extraction kit and amplified by 20-STR amplification. The STR locus and the sex gene Amelogenin were detected on the ABI 3730 genetic analyzer.

Mycoplasma contamination

The cell line is tested negative for mycoplasma contamination.

Commonly misidentified lines
(See ICLAC register)

None

Animals and other research organisms

Policy information about studies involving animals; ARRIVE guidelines recommended for reporting animal research, and Sex and Gender in Research

Laboratory animals

Transgenic mice, such as PDGFRβ-CreER (Jax stock: 030201), NG2-CreER (Jax stock no: 008538), Myh11-CreER (Jax stock no: 019079) , Col1a2-CreER (Jax stock no: 029567) and Rosa26-tdtomato cre reporter line (Jax stock no: 007909) were obtained from the Jackson Laboratory. We developed a new Crabp2-CreER mouse line by inserting CreER fragments into the stop codon of the last exon. All mice used underwent experiments at 2 months-4 months. Both female and male mice were used.
Ten female rhesus macaques (Macaca mulatta) aged 4–7 years old were housed in Beijing Institute of Xieerxin Biology Resource.

Wild animals	Wild animals were not used in this work.
Reporting on sex	Sex was not considered in study design.
Field-collected samples	There were no field-collected samples used in this study
Ethics oversight	All experimental procedures were performed according to Guide for the Care and Use of Lab- oratory Animals which were formulated by the National Institutes of Health (USA) and approved by the Animal Care and Use Committee of the Institute of Genetics and Developmental Biology, Chinese Academy of Sciences. The approved animal protocol number of monkeys is no. 20191017, and the approved animal protocol number of mice is AP2019003 and AP2023031.

Note that full information on the approval of the study protocol must also be provided in the manuscript.

Flow Cytometry

Plots

Confirm that:

- [x] The axis labels state the marker and fluorochrome used (e.g. CD4-FITC).
- [x] The axis scales are clearly visible. Include numbers along axes only for bottom left plot of group (a 'group' is an analysis of identical markers).
- [x] All plots are contour plots with outliers or pseudocolor plots.
- [x] A numerical value for number of cells or percentage (with statistics) is provided.

Methodology

Sample preparation	PDGFRβ-CreER::R26-TdT mice received transection SCI as described above. Ten spinal cord segments (4 mm) per time point centered at the lesion core were isolated and cut into small pieces with sharp scissors. The pieces were digested with papain (Worthington Biochemical, LK003176) containing DNaseI (Worthington Biochemical, LK003170) for 1 h at 37°C with slow rotation. Then 1.0 mg/mL collagenase type II (Worthington Biochemical, LS004176) and 0.4 mg/mL neutral protease (Worthington Biochemical, LS02104) were added, and the sample was incubated for 30 min at at 37°C with slow rotation. After centrifugation at 400g for 5 min, the cell pellets were resuspended with 25% Percoll and centrifuged at 400 g for 10 min to remove myelin debris. Using a BD FACSAria Fusion cell sorter, cells were sorted into DPBS buffer according to tdtomato fluorescence.
Instrument	Flow cytometry was performed by using BD FACSAria Fusion cell sorter
Software	BD FACSDiva 7.0
Cell population abundance	The ratio of fibroblasts and pericytes within post-sort cell suspensions from uninjured spinal cords was higher than that of injured spinal cords. After SCI, other cell types, mainly macrophages/microglia and neutrophil, expressed lower levels of Tdtomato than targeted cells and were sorted out.
Gating strategy	The FSC/SSC was adjusted to isolate single cells, then isolate target cells depending on the expression of Tdtomato.

- [x] Tick this box to confirm that a figure exemplifying the gating strategy is provided in the Supplementary Information.
