# Supplementary material for: Heterogeneous fibroblasts contribute to fibrotic scar formation after spinal cord injury in mice and monkeys
Source: Nat Commun. 2024 Jul 27;15:6321. doi: 10.1038/s41467-024-50564-x (PMC11282111; doi:10.1038/s41467-024-50564-x)
Supplement: Supplementary file 2 — Peer Review File [file 41467_2024_50564_MOESM2_ESM.pdf]

Peer Review File

Heterogeneous fibroblasts contribute to fibrotic scar formation after spinal cord injury in mice and monkey

Open Access This file is licensed under a Creative Commons Attribution 4.0 International License, which permits use, sharing, adaptation, distribution and reproduction in any medium or format, as long as you give appropriate credit to the original author(s) and the source, provide a link to the Creative Commons license, and indicate if changes were made. In the cases where the authors are anonymous, such as is the case for the reports of anonymous peer reviewers, author attribution should be to 'Anonymous Referee' followed by a clear attribution to the source work. The images or other third party material in this file are included in the article's Creative Commons license, unless indicated otherwise in a credit line to the material. If material is not included in the article's Creative Commons license and your intended use is not permitted by statutory regulation or exceeds the permitted use, you will need to obtain permission directly from the copyright holder. To view a copy of this license, visit http://creativecommons.org/licenses/by/4.0/.

Editorial Note: This manuscript has been previously reviewed at another journal that is not operating a transparent peer review scheme. This document only contains reviewer comments and rebuttal letters for versions considered at *Nature Communications*.

REVIEWERS' COMMENTS

Reviewer #1 (Remarks to the Author):

The authors have addressed all my remaining concerns.

Reviewer #2 (Remarks to the Author):

The authors have done a great job addressing my prior critiques, I particularly appreciate their new analysis of fibroblast clusters using recently published mouse brain leptomeningeal fibroblast single cell data. Overall this is a very well done and important study on fibrotic scar origins and heterogeneity in CNS injury.
